# Condensed tannins, novel compounds and sources of variation determine the antiparasitic activity of Nordic conifer bark against gastrointestinal nematodes

**DOI:** 10.1038/s41598-023-38476-0

**Published:** 2023-08-18

**Authors:** Caroline Chylinski, Kristin Fløgstad Degnes, Inga Marie Aasen, Sokratis Ptochos, Berit Marie Blomstrand, Karl-Christian Mahnert, Heidi Larsen Enemark, Stig Milan Thamsborg, Håvard Steinshamn, Spiridoula Athanasiadou

**Affiliations:** 1 Animal and Veterinary Sciences, Scotland’s Rural College, Easter Bush, Roslin, EH25 9RG UK; 2 Department of Biotechnology and Nanomedicine, SINTEF, 7465 Trondheim, Norway; 3 Department of Animal Health, Animal Welfare and Food Safety, Norwegian Veterinary Institute, 1433 Ås, Norway; 4 Norwegian Centre for Organic Agriculture, Gunnarsveg 6, 6630 Tingvoll, Norway; 5 Norwegian Institute of Wood Technology, 0373 Oslo, Norway; 6 Veterinary Parasitology, University of Copenhagen, Dyrlægevej 100, 1870 Frederiksberg, Denmark; 7 Division of Food Production and Society, Grasslands and Livestock, Norwegian Institute of Bioeconomy Research, 6630 Tingvoll, Norway

**Keywords:** Plant sciences, Systems biology, Ecology, Environmental sciences, Diseases, Chemistry

## Abstract

The antiparasitic potential of plants could offer a vital solution to alleviating the costs of gastrointestinal nematode (GIN) infections in ruminant production globally. Leveraging known bioactive molecules, however, is complex, where plant species, extraction processes and seasonality impact bioavailability and efficacy. This study assessed the impact of a comprehensive set of factors on the antiparasitic activity of Norwegian conifers to identify bark compounds specific against GIN. Antiparasitic activity was determined using in vitro assays targeting morphologically distinct life stages of ovine GIN: the egg hatch assay and larval motility assay. In depth characterisation of the chemical composition of the bark extracts was carried out using chromatographic separation, UV-absorbance, and molecular mass profiles to identify compounds implicated in the activity. Three key findings emerged: (1) the activity of bark extracts varied markedly from 0 to 100% antiparasitic efficacy, owing to tree species, extraction solvent and seasonality; (2) the GIN exhibited species-and stage-specific susceptibility to the bark extracts; (3) the presence of condensed tannins, amongst other compounds, was associated with anthelmintic activity. These findings add new insights into urgently needed alternative parasite control strategies in livestock.

## Introduction

Gastrointestinal nematode (GIN) infections are a key threat to the health and productivity of small ruminant farming systems throughout the globe. With widespread resistance against all major antiparasitic drug classes, the current parasite control situation is tenuous. No new drug families have reached the livestock market since monepantel (trade name Zolvix®) was launched in 2009^1^ and novel compounds are urgently needed. Increasingly, antiparasitic discovery is looking towards biopharmaceuticals, including the broad repository of plants that produce antiparasitic molecules^2^. A group of plant secondary metabolites known as condensed tannins (CT) are of particular interest; several CT-rich plants have exhibited high antiparasitic activity against key GIN stages in vitro^3^. In vivo studies using CT-rich forages, such as *Sericea lespedeza* and *Onobrychis viciifolia* Scop., have further demonstrated that moderate consumption by small ruminants can reduce GIN burden^4,5^.

The opportunity to cultivate such forages can, however, be limited in regions with cooler climates, such as the Scandinavian countries. Instead, the bark of Scandinavian coniferous trees has been documented to contain CT^6^ and the antiparasitic potential of bark extracts has recently been demonstrated in vitro^7^. For countries with a substantial sawmill industry where bark is a by-product, the CT contained within may offer a novel GIN control solution as a feed additive, while reinforcing the circularity of the local economy. Recent findings documented considerable variability in the antiparasitic activity of bark extracts, as a function of different tree species, their age at processing, CT type and relative CT content against the ovine GIN *Teladorsagia circumcincta*^7^. The production of specific plant compounds has also been found to vary across a range of other factors including rainfall, soil, temperature, use of pesticides, etc.^8^, with their relative bioavailability depending on the extraction process used^9^; all of these are having an impact on compound composition in bark extracts, which may influence their antiparasitic activity.

In bark, a small part of the CT is free and can be extracted, and these CT constitute only a small part of the total extractives^6,7,10^. Polar solvents, such as water and mixtures of water and water-miscible solvents like methanol, ethanol, or acetone, are used to extract CT. These solvents also extract other polyphenols, including hydrolysable tannins and a wide range of other flavonoids, some of which may be bioactive against parasites. Chemical characterisation of bark extracts is often limited to analyses of total phenolics, CT, sugars etc., but a more comprehensive characterisation of the complex extracts can be obtained by liquid chromatography coupled with mass spectrometry (LC–MS), as recently reported for bark of Norway spruce (*Picea abies*)^11^ and African birch (*Anogeissus leiocarpus*)^12^. Yet, correlating bioactivity to specific compounds in the chemical profile can be challenging, where more than one compound, or synergies between compounds, may be responsible for the observed effects^13^.

The present study aimed to build upon results obtained by Athanasiadou et al.^7^ by assessing the impact of a comprehensive set of factors on the antiparasitic activity of Norway spruce and Scots pine bark extracts from Norway, including collection season, extraction solvent, and concentration. The bark extracts were characterized, and bark compound abundance was quantified using LC–UV and LC–MS analyses. Their relative antiparasitic activity was assessed in vitro against both the eggs and the infective larval stages of two economically important ovine GIN species, *Trichostrongylus colubriformis* and *T. circumcincta*. The associations between compound abundance and antiparasitic efficacy have resulted in identification of candidate compounds in bark and provided new insights in the potential use of plants in the sustainable control of GIN.

## Materials and methods

### Bark sampling and preparation of bark extracts

The use of plant parts in the study complies with international, national, and/or institutional guidelines. Six batches of bark were collected over two collection periods: summer (S), covering July–August 2017, and winter (W), covering February- March 2018. Bark from Norway spruce (*P. abies* L.) was sampled from a sawmill (S1) and a pulp mill (S2), while bark from Scots pine (*P. sylvestris L*.) (P) was from a sawmill. In the pulp mill, the logs were sprayed with cold water prior to debarking. Water was then removed from the bark by pressing, before the bark was shredded. The debarking process at the sawmills did not include water, and the bark was not shredded. Each batch contained equivalent amounts of bark from three partial samples. The partial samples were collected at the mills' debarking plant once a week for a three-week period. About 10 kg of each batch were stored in plastic bags and frozen at − 15 °C. The six batches were abbreviated to reflect the tree species (spruce or pine), mill (1 or 2) and season (summer or winter) and referred to as spruce1-summer (S1-S), spruce1-winter (S1-W), spruce2-summer (S2-S), spruce2-winter (S2-W), pine-summer (P-S) and pine-winter (P-W).

The moisture content of the bark master batches (MB) was determined according to ISO/TS 18,134-2 (2017)^14^. Five samples of bark from each MB were dried in a laboratory oven at 105 °C until the change in mass of the bark did not exceed 0.2% in a heating period of 60 min. The moisture content (M_ar_) of the bark, expressed as percentage of mass, was calculated according to the following formula:

$$M_{ar} = \frac{{\left( {m_{2} - m_{3} } \right)}}{{\left( {m_{2} - m_{1} } \right)}} \times 100$$

m_1_: mass of empty container [g], m_2_: mass of container and the sample before drying [g], m_3_: mass of container and sample after drying [g]

The wood percentage of the bark was determined according to SCAN-CM 53:94 (1994)^15^. Four litres of bark from the moisture content determination were spread on a table. Bark and wood were separated manually and collected in aluminium containers. Wood attached to the bark was separated with a knife. Subsequently, the containers were placed in an oven at 105 °C for 24 h. The wood percentage (W_P_), expressed as percentage of mass, was calculated according to the following formula:

$$W_{P} = \frac{{\left( {m_{2} - m_{1} } \right)}}{{\left( {m_{2} - m_{1} } \right) + \left( {m_{4} - m_{3} } \right)}} \times 100$$

m_1_: mass of containers without wood [g], m_2_: mass of containers filled with wood [g], m_3_: mass of containers without bark [g], m_4_: mass of containers filled with bark[g]

For preparation of the bark extracts, the bark was milled, and extracts were produced as described by^16^ using three different solvents: water, 70% acetone in water (Ace-Wa), and 80% methanol in water (Met-Wa). This produced three different extracts from each of the six bark batches mentioned above and included in the bark code with the addition of -water, -acetone and -methanol. Prior to use, the dried extracts were reconstituted in 50% dimethyl sulfoxide (DMSO) in phosphate buffered saline (PBS), vortexed > 1 min, and diluted with PBS to reach a final extract concentration of 1000, 500, 250, 125, and 62.5 µg/ml in 1% DMSO.

### Quantification and characterisation of condensed tannins

Total CT were quantified by the butanol-hydrochloric acid (butanol-HCl) assay. The freeze-dried extracts were dissolved in methanol (80% in water) and the analyses were carried out using the conventional reagent without co-solvents, 2.5 h incubation, and absorbance reading at 545 nm, with cyanidin-HCl as standard^17^. The relative contents of procyanidins and prodelphinidins, which are the two main types of CT, the mean degree of polymerisation (mDP), and cis–trans-ratio were determined for the Met-Wa extracts by thiolysis with cysteamine hydrochloride and analysis by High Performance Liquid Chromatography (HPLC)^18^ using an Ascentis express C18 column (15 cm × 2.1 mm, 2.7 µm, Supelco) and a flow rate of 0.3 ml/min.

### Characterization of bark extracts by liquid chromatography—mass spectrometry

The bark extracts were analysed using an Agilent 1200 Series Liquid Chromatography–Mass Spectrometry (LC–MS) system equipped with an Ascentis express C18 column (15 cm × 2.1 mm, 2.7 µm, Supelco) connected to a Diode Array Detector (DAD) and an Agilent 6520 Quadruple Time-of-Flight (QTOF) mass spectrometer. The extract concentration was 1000 µg/ml and the injection volume 3 µl. 25 mM formic acid [A] and acetonitrile [B] were used as mobile phases. The concentration of mobile phase [B] was increased from 0 to 90% over a period of 17 min by the following steps: 0% for the first 3 min, then linear gradients from 0 to 8.5% in 2 min, to 50% in 10 min and to 90% in 2 min. The concentration of [B] was then reduced to 0% over a 0.5 min period. The flow rate was 0.3 ml/min, the autosampler temperature was 6 °C, and the column temperature was maintained at 30 °C. The QTOF was equipped with an electrospray- or a Jetstream ionization source operated in positive mode. The gas temperature was set to 345 °C, drying gas flow was 9 L/min, nebulizer pressure 45 psi, fragmentor voltage 150 V, skimmer 47 V and capillary voltage 4000 V. Data was processed with MassHunter qualitative analysis B.06.00 and MassProfiler professional 12.6.1. Putative annotations of identified masses were obtained by automatic annotation or manual search using the Metlin PLCL database. For all putative annotations, the observed isotopic distributions were compared with the theoretical distribution.

### Egg hatch assay (EHA)

The activity of the bark extracts against GIN egg hatching was tested using an EHA, as described in^20^. In brief, GIN eggs were isolated from freshly collected faeces of donor sheep, mono-specifically infected with either *T. colubriformis* or *T. circumcincta* using a flotation technique^19,21^. Eggs were washed with distilled water to remove debris, quantified, adjusted to obtain 100–150 eggs in 250 µl, then added to the wells of a 96-well plate. Control wells received 6 µl of distilled water, and bark treatments received 6 µl of the relevant bark extract, tested at the following concentrations in each well: 1000, 500, 250, 125 and 62.5 µg/mL. Within each assay, the controls and bark extracts were tested in triplicate. The plates were incubated at 20 °C for 48 h. Hatching was stopped by adding a drop of helminthological iodine (10 g iodine, 50 g potassium iodine (KI), 100 ml deionized water in a ¼ dilution) to the samples. The number of eggs and first-stage larvae (L1) present in each well were counted under an inverted microscope at 100 × magnification. The percentage of egg hatching inhibition ($$x)$$, for each bark extract was calculated as:

$${\varvec{x}} = \user2{ }\left( {\frac{{\varvec{a}}}{{{\varvec{a}} + {\varvec{b}}}}} \right)\user2{ } \times 100$$

$x$ is the percentage of unhatched eggs, $a$ is number of eggs and $b$ is the number of L1 in the well.

### Larval motility assay (LMA)

The activity of the bark extracts against L3 (third stage) larval motility was measured using a high-throughput LMA, as described in^7^. In brief, the DP xCELLigence Real Time Cell Analyzer, which measures the electrical impedance-based signals across interdigitated microelectrodes integrated on the bottom of tissue culture e-plates, had previously been adapted to diagnose antiparasitic resistance^22^. Prior to the addition of L3, 50 µl of a 50% phosphate-buffered saline (PBS) in distilled water solution was added to each well for calibration of the assay. The L3 were recovered from faecal cultures of donor sheep, mono-specifically infected with either *T. colubriformis* or *T. circumcincta*, after a 10-day incubation period at 23 °C and extracted using the Baermann technique^23^. The larval suspension was washed twice in the 50% PBS solution and adjusted to obtain 3000 L3 in 146 µl which were then added to each well of the e-plate. Three controls were included in each assay: i) technical control, using a 50% DMSO in PBS solution (1% DMSO final solution); ii) positive control, with alive L3 (1% DMSO final solution) and iii) negative control, with dead L3 (1% DMSO final solution). The dead L3 larvae were obtained by incubating them for 30 min in a 1/10 dilution of 2% sodium hypochlorite sterilising fluid, followed by repeated washing in 50% PBS in distilled water. A light-proof box was placed over the e-plates to exclude potential light interference on L3 motility. The impedance was recorded for each well every 15 s, for 24 h, at 20 °C. Twenty-four hours after the assay began, 4 µl of the bark extracts were added to each well, tested at a single concentration of 1000 µg /ml . Positive controls received 4 µl of 50% DMSO in PBS solution, whereas negative controls received 4 µl of the bark extracts in each well. All bark treatments and controls were tested in triplicate. For each GIN species, all extracts were tested in a single assay. Impedance data were converted into a motility index based on the curve scatter as described by^22^ prior to statistical analysis.

### Statistical analyses

EHA data: Prior to analysis, the residuals of all egg hatch data were tested, and normality was confirmed. Data for each of the GIN species were analysed separately using the software package Genstat (Version 18; VSN international, 2020). Significant differences in the data were tested using ANOVA, including interactions between each of the variables in the bark extracts. The extract concentration required to inhibit 50% hatching (IC50) for each of the bark extracts was calculated using probit analysis. A targeted, Pearson correlation analysis was performed using the ‘corr’ procedure in SAS (SAS release 9.4, SAS Institute, Cary, NC) to associate CT content in the extracts to the IC50-values for each GIN species.

LMA data: Larval motility was measured for 24 h prior to the addition of the bark extracts, and for 24 h following the addition of the extracts (48 h total). To quantify the antiparasitic activity of the extracts, two 6-hour windows were selected: 15–21 h after the start of the assay, and 15–21 h following the addition of the bark extracts to the wells. These 6-hour windows were selected to minimise any potential impact of the assay procedures (e.g., addition of extracts) on larvae motility readings. Motility data following the addition of the bark extract were analysed using multiple comparison ANOVA, with bark, solvent and season included in the model as factors. Motility data prior to the addition of the bark extracts were used as a covariate. Bonferroni multiple comparison correction threshold was set at *P* < 0.05. Statistical analyses were carried out separately for each GIN species; the null hypothesis tested was that the motility of the larvae exposed to bark extracts was significantly different (*P* < 0.05) from the motility of the dead control larvae, thus a significant effect (i.e. when the null hypothesis is rejected) would indicate a strong antiparasitic activity from the specific bark extract. Previous experience indicated that the variation of the three technical replicates is minimal in this assay^7^.

Principal component analysis (PCA) of mass spectroscopy data was performed to characterise the compound variation between the extracts. PCA plots were produced using Mass Profiler Professional, version 12.6.1, Agilent Technologies. Following the identification of bark compounds with LC–MS, a Pearson correlation analysis was performed to identify potential candidate-compounds responsible for the observed anthelmintic activity. To achieve this, the log transformed abundance of each of the LC–MS determined masses in the extracts was associated with the inhibition (IC50) of *T. colubriformis and* T*. circumcincta* egg hatching.

## Results

### Season, tree species and solvent contribute to the variation observed in the CT yield and concentration in bark samples

The CT extraction yields varied between 2 and 16 mg/g dry bark, and the CT concentration in each extract ranged from 17 to 153 mg/g extract DM (Table 1). Overall, Ace-Wa extracted the greatest concentration of CT. The water and Ace-Wa pine extracts had lower CT yields and CT concentrations than the two spruces. Further, only pine extracts had higher CT concentration in summer than winter, irrespective of the solvent used in the extraction. Of note, the pine also contained the highest (winter) and lowest (summer) wood content compared to the other barks (Supplementary Table S1). Of the two spruce barks, spruce2 (S2), which originated from a pulp mill and used water in the debarking process, had lower DM-yields and CT-concentrations than spruce1 (S1), which came from a sawmill where water was not used. The mDP for the CT of the Met-Wa extracts was similar for all bark sources and varied in the range 5.7–7.8. Prodelphinidins were not detected in the pine bark extracts (100% procyanidins) and constituted 2–3% of the total CTs in the spruce extracts (97–98% procyanidins). The *cis*-isomer (epicatechin) constituted 87% for spruce and 79–83% for pine (lowest in summer).

**Table 1:** Yield and concentration of condensed tannins (CT) in water, acetone–water and methanol–water extracts of bark from different tree species, origin and season.

Bark	Solvent	CT yield per gram dry bark (mg/g bark)		CT concentration in extracts (mg/g extract DM)	
		Summer	Winter	Summer	Winter
S1	Water	2.7	8.7	70	80
S2		2.5	3.5	17	48
P		1.5	1.8	65	33
S1	Acetone–water	11.0	15.7	107	122
S2		7.8	8.7	91	101
P		5.4	5.3	153	95
S1	Methanol–water	5.2	10.3	98	106
S2		3.6	3.8	68	68
P		3.8	4.2	142	91

Quantification was carried out on bark extracts at the highest concentration tested in the GIN assays (1000 µg extract/mL). Bark extracts include: (S1) spruce, sawmill, ring debarking; (S2): spruce, pulp mill, drum debarking; (P): pine, sawmill, ring debarking. Season bark was collected, summer and winter.

### CT content significantly associated with a reduction in *T. colubriformis* egg hatching, but not in* T. circumcincta*

A proportion of the control eggs did not hatch for both *T. colubriformis* (14%) and *T. circumcincta* (8%) (Table 2). Most of the bark extracts significantly inhibited egg hatching for both GIN, when tested at the top concentration. All bark extracts that were highly effective or effective against *T. circumcincta* showed a similar pattern of efficacy also against *T. colubriformis*; in general, the latter were more susceptible to inhibition against a greater range of extracts, and at lower concentrations. For the majority of Ace-Wa and Met-Wa extracts a dose response was evident, whereas there was little or no evidence of a dose response for the water extracts.

**Table 2:** Mean egg hatch inhibition (%) results from bark extracts against two ovine GIN species.

		Solvent	Water					Acetone–water					Methanol–water					Control
GIN	Bark	Season	1000	500	250	125	62.5	1000	500	250	125	62.5	1000	500	250	125	62.5	
*T. colubriformis*	S1	S	**25**	**16**	**15**	12	14	***100***	***100***	**54**	**28**	**24**	***100***	*95*	**56**	**18**	13	14
		W	**32**	**20**	**26**	**15**	**15**	***100***	***100***	**85**	**35**	12	***100***	*91*	**37**	**22**	**20**	
	S2	S	10	11	**16**	11	7	***100***	*93*	**69**	**34**	**16**	***100***	*91*	**50**	**29**	14	
		W	**59**	**40**	**21**	**18**	**21**	***100***	***100***	***99***	**56**	**30**	***100***	***100***	**68**	**29**	**25**	
	P	S	**34**	**28**	**29**	**14**	11	***100***	***100***	*96*	**74**	**26**	***100***	*97*	*95*	**50**	**39**	
		W	**59**	**55**	**40**	**22**	**18**	***100***	***100***	***100***	***100***	*94*	***100***	***100***	***100***	*92*	**50**	
*T. circumcincta*	S1	S	7	6	2	4	2	**62**	4	2	4	1	**11**	4	5	3	1	8
		W	**38**	**21**	**15**	**16**	**15**	*98*	**56**	**14**	**14**	**19**	*97*	**28**	**17**	**10**	**22**	
	S2	S	4	4	5	3	5	**25**	5	3	4	3	**16**	3	4	4	2	
		W	**63**	**18**	**15**	**13**	**18**	***100***	*96*	**27**	**20**	**18**	***100***	**39**	**15**	**16**	**16**	
	P	S	**35**	4	3	4	4	*95*	**44**	5	4	3	*93*	**24**	2	4	3	
		W	**85**	**43**	**20**	**16**	**13**	***100***	***100***	***100***	**35**	**20**	***10 0***	*94*	**60**	**22**	**14**	

Mean egg hatch inhibition (%) of bark extracts (n = 3) tested against both *T. colubriformis* and *T. circumcincta* eggs. S1: spruce, sawmill, ring debarking; S2: spruce, pulp mill, drum debarking; P: pine, sawmill, ring debarking. Collection seasons from summer (S) and winter (W). Results in bold were significantly different from the control treatments (one-way ANOVA and post-hoc Bonferroni test for multiple comparisons, where *P* ≤ 0.05). Results highlighted in bold italic were considered highly active (≥ 99% inhibition), and those in italic considered active (90–98% inhibition).

Of the variables associated with the anti-parasitic activity in the EHA, there was a significant effect of the tree species, (*P* < 0.001), season (*P* < 0.001), solvent (*P* < 0.001) and GIN species (*P* < 0.001) (Table 3), with significant interactions observed between GIN and solvent (*P* < 0.001), GIN and season (*P* < 0.001), bark and season (*P* = 0.042). Pine was more effective than either of the spruces against both GIN species, but the two spruces did not differ significantly. Overall, bark collected in winter was more effective than the summer. The Met-Wa and Ace-Wa extracts were more effective than water against *T. colubriformis* eggs. Similarly, Met-Wa extracts were more effective against *T. circumcincta* eggs than water extracts, but the impact of Ace-Wa extracts did not differ significantly from either of the other extracts.

**Table 3:** Impact of bark extract variables on the anti-parasitic activity observed in egg hatch assay (EHA) against two ovine GIN species.

Variable	*T. colubriformis*		*T. circumcincta*	
	*p* value	Significance	*p* value	Significance
Tree species	< .001	S1^b^, S2^b^, P^a^	< .001	S1^b^, S2^b^, P^a^
Season	0.025	S^b^, W^a^	< .001	S^b^, W^a^
Solvent	< .001	Wa^b^, Ace-Wa^a^, Met-Wa^a^	< .001	Wa^b^, Ace-Wa^ab^, Met-Wa^a^
Concentration	< .001	1000^a^, 500^ab^, 250^b^, 125^c^, 62^c^	< .001	1000^a^, 500^b^, 250^c^, 125^c^, 62^c^

Variables of bark extracts tested within GIN species included: Bark (S1): spruce, sawmill, ring debarking; (S2): spruce, pulp mill, drum debarking; (P): pine, sawmill, ring debarking; Season summer (S) and winter (W); solvent used in extraction (Wa) water, (Ace-Wa) acetone–water, (Met-Wa) methanol–water; tested at five different concentrations (µg DW/mL). Significant differences (one-way ANOVA, *P* ≤ 0.05) denoted with letters in superscript, where ^a^ > ^b^ > ^c^.

The pine-winter-Ace-Wa extract showed the highest efficacy at the lowest concentration against both GIN species, which was lower in *T. colubriformis* than *T. circumcincta* (100% efficacy at 125 µg DM/ml and 250 µg DM/ml, respectively) (Table 2). Indeed, the pine-winter-acetone extract had the lowest lethal dose in both GIN species, which was almost four-fold lower in *T. colubriformis* (48 µg DM/ml) than *T. circumcincta* (221 µg DM/ml) (Table 4). In *T. circumcincta*, spruce2-winter-Ace-Wa had an equally low IC50 (221 µg DM/ml); the IC50 of the other bark extracts were considerably higher.

**Table 4:** IC50 of the 18 bark extracts to inhibit egg hatching, ordered from the lowest to the highest concentration against two ovine GIN species.

*T. colubriformis*				*T. circumcincta*			
Bark	Season	Solvent	IC50 (µg DM/mL)	Bark	Season	Solvent	IC50 (µg DM/mL)
P	W	Ace-Wa	48	P	W	Ace-Wa	221
P	W	Met-Wa	63	S2	W	Ace-Wa	221
P	S	Ace-Wa	89	S1	W	Ace-Wa	353
P	S	Met-Wa	93	S2	W	Met-Wa	407
S2	W	Ace-Wa	95	P	W	Met-Wa	407
S2	W	Met-Wa	145	P	W	Wa	459
S2	S	Ace-Wa	159	S1	W	Met-Wa	475
S1	W	Ace-Wa	162	P	S	Ace-Wa	492
S2	S	Met-Wa	193	P	S	Met-Wa	596
S1	S	Met-Wa	196	S1	S	Ace-Wa	1050
S1	W	Met-Wa	208	S2	W	Wa	1282
S1	S	Ace-Wa	400	P	S	Wa	3587
P	W	Wa	495	S2	S	Ace-Wa	5908
S2	W	Wa	856	S1	W	Wa	7093
P	S	Wa	3,498	S2	S	Met-Wa	20,733
S1	W	Wa	16,203	S1	S	Met-Wa	83,393
S1	S	Wa	212,179	S1	S	Wa	4,236,058
S2	S	Wa	1.8^14^	S2	S	Wa	1.2^11^

IC50: lethality dose required to inhibit 50% of the GIN eggs from hatching. Calculated based on results from dose–response egg hatch assay (EHA). Bark extracts tested included: (S1): spruce, sawmill, ring debarking; (S2): spruce, pulp mill, drum debarking; (P): pine, sawmill, ring debarking and were collected during summer (S) and winter (W) seasons. Each of the barks were extracted using water (W), acetone–water (Ace-Wa), and methanol–water (Met-Wa) as the solvent.

The targeted Pearson correlation revealed a significant negative correlation between the CT content in the different bark extracts and the *T. colubriformis* egg hatching inhibition (IC50) r = − 0.54 (*P* < 0.021), while the respective correlation for *T. circumcincta* was not significant (r = − 0.14, *P* = 0.579).

### Water bark extracts were the most efficient in reducing the motility of L3 in ovine GIN

Throughout the LMA assays, the motility of the alive control larvae was consistently higher than that of the dead control larvae. Motility values of alive larvae were not included in the statistical analysis but were only used as technical controls. Our data showed that six out of the 18 bark extracts reduced the motility of *T. colubriformis* larvae to that of the dead controls: four water and two Met-Wa-based extracts (Table 5). For *T. circumcincta,* our data showed that larvae motility was reduced to that of dead controls by four water, four Ace-Wa, and three Met-Wa-based extracts. In some cases, motility of larvae following the addition of the extract was lower compared to that of the alive controls, but still significantly different from dead controls.

**Table 5:** L3 larvae motility following incubation with bark extracts at 1000 µg/mL dry weight in two GIN species.

		Solvent	Water				Ace-Wa				Met-Wa			
GIN species	Bark	Season	Alive	Dead	Bark	H_0_ Rejected	Alive	Dead	Bark	H_0_ Rejected	Alive	Dead	Bark	H_0_ Rejected
*T.colubriformis*	S1	**S**	**0.0043**	**0.0022**	**0.0035**	**Y**	0.0043	0.0022	0.0042	N	**0.0043**	**0.0022**	**0.0038**	**Y**
		**W**	**0.0043**	**0.0022**	**0.0037**	**Y**	0.0043	0.0022	0.0040	N	**0.0043**	**0.0022**	**0.0039**	**Y**
	S2	S	0.0042	0.0008	0.0037	N	0.0042	0.0008	0.0038	N	0.0042	0.0008	0.0038	N
		W	0.0042	0.0008	0.0037	N	0.0042	0.0008	0.0041	N	0.0042	0.0008	0.0040	N
	P	**S**	**0.0043**	**0.0009**	**0.0033**	**Y**	0.0043	0.0009	0.0044	N	0.0043	0.0009	0.0042	N
		**W**	**0.0043**	**0.0009**	**0.0033**	**Y**	0.0043	0.0009	0.0035	N	0.0043	0.0009	0.0046	N
*T.circumcincta*	S1	**S**	**0.0088**	**0.0038**	**0.0038**	**Y**	**0.0088**	**0.0038**	**0.0041**	**Y**	**0.0088**	**0.0038**	**0.0040**	**Y**
		**W**	**0.0088**	**0.0038**	**0.0042**	**Y**	**0.0088**	**0.0038**	**0.0039**	**Y**	**0.0088**	**0.0038**	**0.0041**	**Y**
	S2	**S**	**0.0077**	**0.0031**	**0.0040**	**Y**	0.0077	0.0031	0.0041	N	0.0077	0.0031	0.0041	N
		W	0.0077	0.0031	0.0045	N	**0.0077**	**0.0031**	**0.0038**	**Y**	0.0077	0.0031	0.0046	N
	P	**S**	**0.0077**	**0.0048**	**0.0067**	**Y**	**0.0077**	**0.0048**	**0.0038**	**Y**	**0.0077**	**0.0048**	**0.0041**	**Y**
		W	0.0077	0.0048	0.0043	N	0.0077	0.0048	0.0042	N	0.0077	0.0048	0.0047	N

Values for the ‘alive’ L3 control, ‘dead’ L3 control and ‘bark’ treated L3 represent mean (n = 3) larval motility adjusted for the covariate (motility of larvae prior to the addition of bark extracts). Bark treatments included: S1: spruce, sawmill, ring debarking; S2: spruce, pulp mill, drum debarking; P: pine, sawmill, ring debarking and were collected during summer (S) and winter (W) seasons. Each of the bark samples was extracted using water, acetone–water (Ace-Wa), or methanol–water (Met-Wa) as the solvent. The null hypothesis (H_0_) tested was that the motility of the larvae in the bark extract treatment differed significantly from the dead controls; rejected null hypothesis (Y) was indicative of strong antiparasitic activity, whereas not-rejected null hypothesis (N) was indicative of no strong antiparasitic activity. Bold are the extracts that showed significant antiparasitic activity.

Four of these extracts demonstrated strong anthelmintic efficacy against larvae of both GIN. Three of these were from spruce (S1) and one from pine (P). In both GIN species, water extracts were more frequently effective at reducing larvae motility at the level of control dead larvae (8 extracts), with Met-Wa extracts coming second (4 extracts) and the Ace-Wa extracts being the least effective (2 extracts) in reducing the motility of larvae to that of dead larvae.

### Significant variation in the compound profiles of the bark extracts tested by liquid chromatography—UV—mass spectrometry

**Figure 1:**
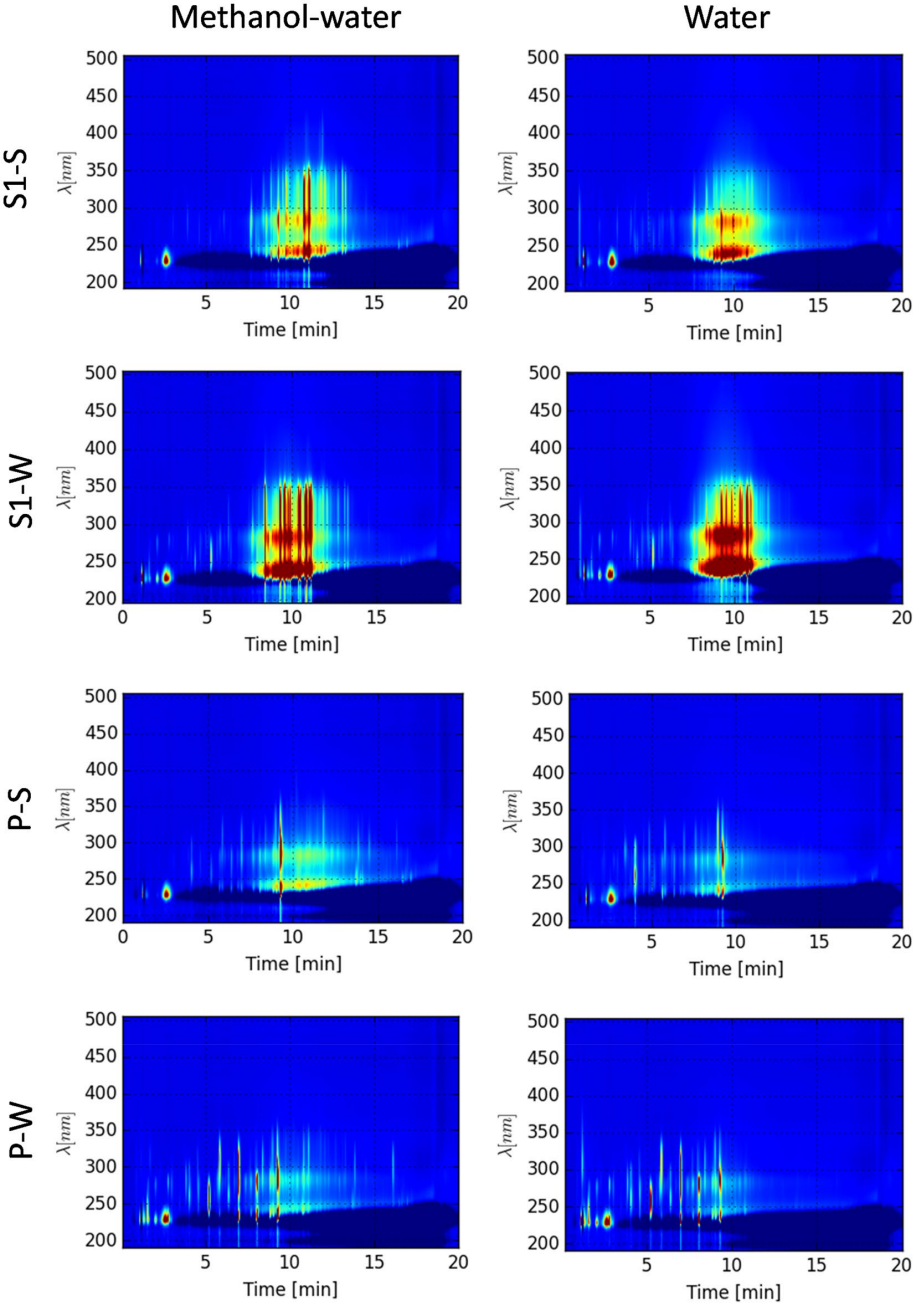
LC-DAD-isoplots of 8 bark extracts, which represent all main UV peaks observed among the 18 analysed extracts. The analysis was performed with reverse phase chromatography, where hydrophilic compounds elute first and the more the hydrophobic compounds at higher retention times. UV wavelengths is shown on the y-axis and the high-performance liquid chromatography (HPLC) retention time on the x-axis. The colour represents a relative concentration of the UV absorbing compounds where red = high concentration, yellow = medium concentration, blue = low concentration/absence. S1-S: spruce1 summer; S1-W: spruce1 winter; P-S: pine summer; P-W: pine winter.

The LC-DAD-MS-analyses were performed with a diode array detector (UV) connected upstream of the MS. The UV-absorbance of the extracts, presented as LC-DAD-isoplots (Fig. 1) and UV chromatograms (280 nm, Supplementary Fig. S1), revealed that the extracts from the two spruce samples, contained the same UV-absorbing compound classes, but with an overall lower concentration of compounds in spruce2. The concentration of UV-absorbing compounds from spruce1 was considerably higher in the bark collected in winter than in summer (The winter extracts from both spruce sources also contained hydrophilic compounds not visible in the summer extracts (Fig. 1, retention time below eight minutes). The lower concentrations of UV absorbing compounds in spruce2 were in accordance with the DM and CT-yields and could be attributed to the use of water during the debarking process. No significant differences could be observed between the LC-DAD-isoplots of the Met-Wa and Ace-Wa extracts (Supplementary Fig. S2), whereas the water extracts contained other UV absorbing compounds than the organic solvent extracts.

The majority of the UV-absorbing compounds in the spruce extracts eluted between 8 and 11 min (Fig. 1). The concentrations of this group of compounds were higher in the winter compared to the summer extracts. This characteristic group of compounds was not present in the pine extracts. Instead, the pine extracts contained UV absorbing compounds that, due to different UV-profile and elution over a wider time range, most likely belong to different chemical groups. In addition, the compounds in the pine Met-Wa and Ace-Wa extracts eluting later than 10 min in the chromatograms, were not extracted with water. By comparing the UV- and MS-data, molecular masses of some of the UV-absorbing compounds of the extracts could be identified and the compounds putatively annotated. The UV-absorbing compounds from spruce bark eluting between 8 and 11 min all had UV spectra similar to flavones (Table 6).

**Table 6:** UV-absorbing compounds observed in extracts of only one of the two tree species based on the spectra presented in Fig. 1 and Supplementary Fig. S2. The compounds were putatively assigned to compound classes based on MS- and UV spectra and retention times.

Extract source	Retention time[min]	Monoisotopic mass (Da)	Putative annotation (Compound or chemical group)	UV maxima (nm)
Spruce^1^	8.53	488.1457	Flavones and substituted flavones, eg. flavone glycosides	234, 289, 304, 323
	8.54	423.1521		234, 289, 304, 323
	9.41	456.1564		232, 287, 305, 321
	11.03	504.1445		235, 288, 306, 322
	11.15	514.1675	trihydroxy-flavan-dimer	238, 290, 302, 325
Pine	5.8	146.0367	Coumarin	237, 294, 311
	7.0	218.0554 and 507.2309	Coumarin derivatives	231, 278, 305
	16.2	226.1004	Pinosylvin monomethyl ether^2^	249, 299

^1^Annotated based on UV-spectra^21–23^ and occurrence only in the spruce bark extracts.

^2^Annotated based on^24^ and occurrence only in pine.

Whilst UV analysis demonstrated that the extracts tested showed differences in 20–50 compounds, the MS analysis detected 2299 different compounds in the 18 extracts. In agreement with the UV data, the extracts from bark collected in winter contained more compounds than bark harvested in the summer. This was particularly evident for the water extracts from pine, where 480 compounds were detected in the winter sample compared to 248 compounds in the summer one (Supplementary Figure S3). For both spruce bark sources, Met-Wa extracts showed the highest number of masses whereas for pine, the Ace-Wa extract showed the highest compound diversity. The extracts from pine bark contained a higher number of compounds than the bark from both spruces. The total number of compounds detected in the Ace-Wa, Met-Wa, and water extracts of winter samples of spruce1, spruce2 and pine were 1098, 829 and 1511, respectively (Supplementary Figure S3). The PCA of the complete MS data set showed that pine-winter extracts were separated from the other samples (Supplementary Fig. S4). The 97 masses with the strongest contribution to the group separation in the PCA plot (loading > 3 or < -3 for component 1 and/or component 2) of pine-winter extracts, eluted early in the chromatogram, indicating that they are hydrophilic compounds (Supplementary Table S2).

Presence of CT molecules with three or less monomeric units (mDP ≤ 3) were detectable in the MS-analysis, eluting at retention times between 6 and 8 min. They occurred with higher abundance in the pine than in the spruce extracts and were not detected in the spruce2 samples. Three CTs were identified in the extracts from spruce and/or pine with less than 5 parts per million (ppm) mass error and a correlating isotopic distribution. These compounds were procyanidin C1 (CT trimer), procyanidin B1/B2 (CT dimer), and catechin/epicatechin (CT monomer) (Supplementary Table S3).

### LC–MS detected masses significantly associated with inhibition of GIN egg hatching activity

A correlation analysis between the 2299 detected masses and EHA IC50 of *T. colubriformis* and *T. circumcincta* was performed. Since the Ace-Wa and Met-Wa extracts had a higher overall bioactivity than the water extracts, it is not unlikely that different compounds were responsible for the strong effect of the organic solvent extracts and the weaker effect of the water extracts. Therefore, correlation analysis was performed using (1) all extracts and (2) only Met-Wa and Ace-Wa extracts. When all extracts were included in the analysis, only one compound of unknown id (r = − 0.54, *P* = 0.02) was significantly negatively associated with IC50 of *T. colubriformis* egg hatching. When only the Met-Wa and Ace-Wa extracts were included, a total of 69 compounds were significantly negatively associated with *T. colubriformis* egg hatching IC50. Most of the masses correlating with *T. colubriformis* inhibition were detected only in pine. For *T. circumcincta*, four compounds were negatively associated with IC50 on egg hatching when all extracts were included, and five compounds when only Met-Wa and Ace-Wa extracts were included in the correlation analysis. Since the Met-Wa and Ace-Wa extracts showed relatively similar efficacy that was consistently higher than the water extracts, an additional analysis of the Met-Wa extracts were conducted. A total of 1316 compounds were identified in the six Met-Wa extracts (i.e., spruce1-summer, spruce1-winter, spruce2-summer, spruce2-winter, pine-summer, and pine-winter) with this method; importantly, 89 new compounds were identified that we had not identified previously. A Pearson correlation analysis of the detected masses revealed that 46 of these had a significant negative correlation to the IC50 from the egg hatch assay of *T. colubriformis* (Supplementary Table S4).

For a visual assessment of the relationship between the candidate compounds that originated from the statistical analysis and the anthelmintic activity, the relative abundance of each of the candidate masses was plotted against the anthelmintic activity given as 1/IC50. Table 7 and Supplementary Fig. S5 shows this relationship for seven masses regarded as particularly interesting based on this visual inspection. These masses were all correlated to the inhibition of *T. colubriformis*, as this was the GIN that appeared to be most susceptible by the extracts. Two compounds that were found in both pine and spruce with masses 164.0832 and 200.1557 were the only two ones with r > 0.95 and *p* < 0.01 (Table 7). Based on accurate molecular masses and isotopic distribution observed with MS (Supplementary Table S5), the molecular formulas of these compounds are likely to be C_10_H_12_O_2_ and C_15_H_20_, respectively. A high number of benzene ring containing molecules with these formulas exist, and possible annotations for these molecules are eugenol (a monoterpenoid) for the first one, and a sesquiterpenoid (e.g., calacorene) for the other. The mass M = 226.1004 eluted at the retention time that corresponded to a UV peak found in pine extracts (Fig. 1; Tables 6 and 7) and is likely to be pinosylvin monomethyl ether, a stilbene (ref). The mass 300.2089 could potentially be a naphthalene derivative.

**Table 7:** Selected masses where the abundance in the extracts from two independent MS-analyses correlated with the inhibition of egg hatching in *T. colubriformis* (low *p*-value/high r-value).

Monoisotopic mass (Da)	Retention time[min]	Species	Correlation analysis^1)^			
			Met-Wa		Met-Wa and Ace-Wa	
			r	*p*-value	r	*p*-value
164.0832	8.18	Both	− 0.92	0.01	− 0.82	0.001
196.0733	7.05	Pine	0.01		− 0.82	0.001
200.1557	15.4	Both	− 0.99	< .0001		
226.1004	16.24	Pine	− 0.93	0.01		
300.2089	16.7	Both			− 0.71	0.0047
379.1993	10.86	Pine			− 0.82	0.001
380.1093	6.8	Pine	− 0.93	0.01	− 0.72	0.009

^1^The correlation analyses were performed on two independent datasets: Met-Wa: Analyses of the Met-Wa extracts using a jet-stream ion source; Met-Wa and Ace-Wa: Analyses of all extracts using Electrospray ionization (ESI), with the statistical analyses including the datasets for the Met-Wa and Ace-Wa extracts. Complete datasets: Supplementary Table S4.

## Discussion

The objective of this study was to assess the impact of a comprehensive set of factors on the antiparasitic activity of bark extracts from Norwegian conifer trees in vitro and identify compounds that may be associated with the antiparasitic activity of bark. Three key findings emerged: (1) certain bark extracts demonstrated very high antiparasitic activity, inhibiting 100% of eggs from hatching and/or reducing the L3 larvae motility to levels indicative of death. These results confirm the potential of using Norwegian bark extracts in GIN control; (2) variation in susceptibility of the two species tested was evident, with *T. colubriformis* egg hatching being affected more than that of *T. circumcincta*; the opposite was the case for the impact of bark extracts on larvae motility; (3) the presence of CT and numerous other compounds was significantly associated with the observed antiparasitic activity.

The yield and concentration of CT in our bark extracts were similar to those observed in other studies using sawmill by-products^6,7,10^. Overall, the dry matter and CT-yields were lower in extracts from the summer bark, which may be explained by spraying of the logs and leakage of water-soluble compounds. The relatively high CT-content of the pine summer extracts, combined with the lower dry matter, indicate that fewer other compounds were extracted from this bark. The pine-winter bark had the highest wood content, which offers a likely explanation for the high number of compounds detected in the extracts from this bark and the separation in the PCA-plot. As none of these masses were significantly more abundant in the Ace-Wa and Met-Wa extracts than in the water extract, they cannot explain the high bioactivity of the Ace-Wa and Met-Wa extracts from pine. The greater yields obtained by Ace-Wa and Met-Wa compared to water is likely to be attributable to the organic solvents capacity to disrupt bonds to other compounds, thus releasing more of the bound CT^25^, however, differences in solubilities between The CTs may also have contributed.

The characterisation of the chemical composition of the bark extracts that was carried out based on chromatographic separation, UV-absorbance, and molecular mass profiles offered a lot of novel data. The main aim of this characterisation was to explain the differences in bioactivity. However, the analyses also revealed distinct differences between the UV-profiles of the spruce and pine extracts, and the MS-analysis detected 878 compounds that were unique to pine when compared to spruce1, debarked by the same method, while 595 compounds were unique to spruce1, compared with pine. Published LC–MS identification of bark compounds is scarce, but clear differences in the MS-profiles of bark extracts from different conifer tree species, including Norway spruce, Scots pine and Silver fur (*Abies alba*) have been observed^11,24^.

Certain bark extracts inhibited egg hatching of both GIN species up to 100%, although *T. colubriformis* exhibited greater susceptibility to lower concentrations of extracts (IC50) than *T. circumincta.* The associations of EHA IC50 with concentrations of different compounds in extracts showed that CT were at least partly responsible for the *T. colubriformis* egg hatching inhibition (r = − 0.54). These results support that CT play a role in the egg hatching inhibition at least of the *T. colubriformis* eggs. Although some of the variation in the anthelmintic activity against *T. colubriformis* egg hatching is explained with variation in CT content, this is not the case for *T. circumcincta*. This observation is in agreement with previous evidence^7^, where variation in the anthelmintic efficacy of bark extracts was not always associated with overall CT content. Indeed, additional CT characteristics are thought to impact antiparasitic effects of CT, such as the degree of polymerisation and type of monomeric units^26^. The pine and spruce bark contain almost solely procyanidins, but as the thiolysis only captures the mean degree of polymerisation, it cannot be excluded that some of the extracts have larger variation in mDP and thus contain larger CT-molecules. However, since the CT constituted less than 15% of the extract DM, a more likely explanation for the variation in the efficacy observed is contributions from other compounds. In support of this, the association of MS-data with the inhibition of egg hatching in *T. colubriformis* (Table 7, Supplementary Fig. S5) showed other compounds that likely have a role to play in the antiparasitic activity of bark extracts. Several plant secondary metabolites have been related to such activity, including terpenoids, saponins, flavonoids, hydroxycinnamic acid derivatives and other polyphenolic compounds^26,27^, such as flavanol derivatives^28^ which may act synergistically to achieve higher antiparasitic activity^29^. Indeed,^13^ is a good example of verified interactions. The use of polyvinylpolypyrrolidone (PVPP), which blocks CT, has been particularly useful in demonstrating that CT are not solely responsible for antiparasitic activity, and some studies have even observed improved antiparasitic activity following PVPP incubation, indicating potentially antagonistic effects between plant secondary metabolites^30,31^. In our study, that the pine extracts had comparatively lower IC50 than the spruce extracts, indicates that some of the compounds that were absent in the spruce, for example those that have retention time over 14 min (Fig. 1), may be responsible for the antiparasitic activity. However, as both spruces still demonstrated strong antiparasitic activity at higher concentration, it seems possible that different compounds may contribute to the bioactivity of spruce and pine.

In relation to the latter, indeed, highly polar solvents, such as methanol, have been known to release more bioactive compounds from plants compared to water only, which are specific to ovicidal activity, or they may result in releasing fewer compounds which antagonize activity^31,32^. species-specific differences in the susceptibility of GIN to both plant compounds^7,33–35^ and broad-spectrum anthelmintics^36–39^ have been previously documented in vitro, where the pharmacological activity can depend upon interactions with species—specific enzymes, proteins, nucleic acids, biomolecules, and receptors^40^. Similarly, the antiparasitic efficacy of the bark extracts varied depending on the GIN life stage they were targeted against. This is likely owing to the distinct structural and compositional characteristics of the egg membrane compared to the larval cuticle, which may interfere with the compounds mechanism of action and resulting antiparasitic activity^41^. The GIN eggshell is composed of three layers; the inner lipid, medial chitin and outer vitelline^42^. The L3 larvae stage possesses a double cuticle sheath, consisting of four parts: a triple layered epicuticle at the external surface and the inner-most stratum, primarily composed of collagen (insoluble in detergent), soluble proteins and components of low molecular weight, such as lipids^43,44^. It has been suggested that the GIN eggshell is comparatively more permeable to some antiparasitic compounds than the L3 larvae cuticle^44^, however, further studies would be required to establish an IC50 for the L3 larvae motility before any conclusions could be drawn for the bark extracts.

The LC–UV–MS analyses were performed with the main aim to reveal differences between the extracts that could contribute to explain the differences in antiparasitic activities not explainable by the CT-content. The most abundant masses that were strongly correlating with the bioactivity of the extracts, were annotated by search in databases. However, as bark contain a high number of bioactive phenolic compounds with very similar structure^45^, a reliable identification was not possible. This would require MS/MS-analysis or NMR. Nevertheless, two masses were present in both pine and spruce, and were negatively associated with IC50. One mass was putatively annotated as monoterpenoid eugenol, previously reported to significantly reduce the total worm burden in humans^46^. The molecular mass of the other corresponded to several sesquiterpenoids; one of them, calacorene has previously been detected in plant extracts active against the *Onchocerca ochengi*^46^.

In conclusion, this paper has presented abundant in vitro evidence to support the possible exploitability of Norwegian sawmill by-products as a tool in the control of ovine GIN. Importantly, it has identified novel sources of variation associated with antiparasitic activity and specific compounds that warrant further study. This is the first in vitro study conducted on such a scale, where 18 different extracts were studied, and solid associations were made between compounds and efficacy. From the 18 extracts tested, many showed activity against one or more of the stages and species. Although CT showed a significant association with antiparasitic activity against *T. colubriformis,* other compounds have contributed to the activity observed. These findings add new insights to a growing library of bioactive plants that contain key compounds for the future control of ovine GIN in a time of anthelmintic resistance. Additional characterization studies with MS/MS fragmentation and NMR would be necessary to identify the specific compounds that are responsible for the activity. Validation of the results in vivo would further ascertain host tolerance to the bark based on production parameters and indicators of toxicity. Further exploitation will largely depend on the possibility for upscaling; as we present evidence of antiparasitic activity in water extracts, upscaling appears to be a realistic option.

### Supplementary Information

Supplementary Information.

## Data Availability

The datasets used and/or analysed during the current study are available from the corresponding author on reasonable request.
