# Supplementary material for: Condensed tannins, novel compounds and sources of variation determine the antiparasitic activity of Nordic conifer bark against gastrointestinal nematodes
Source: Sci Rep. 2023 Aug 18;13:13498. doi: 10.1038/s41598-023-38476-0 (PMC10439207; doi:10.1038/s41598-023-38476-0)
Supplement: Supplementary file 1 — Supplementary Information. [file 41598_2023_38476_MOESM1_ESM.pdf]

Caroline Chylinski, Kristin Fløgstad Degnes, Inga Marie Aasen, Sokratis Ptochos, Berit Marie Blomstrand, Karl-Christian Mahnert, Heidi Larsen Enemark, Stig Milan Thamsborg, Håvard Steinshamn, Spiridoula Athanasiadou:

The antiparasitic activity of Nordic conifer bark against gastrointestinal nematodes: condensed tannins, novel compounds, and sources of variation

Supplementary Tables and Figures

Supplementary Table S1: Properties of the bark batches.

Bark batch	Wood species	Location Norway	Debarking method	Moisture content (% of wet mass)		Wood percentage (% of dry mass)	
				S	W	S	W
S1	Spruce	Brandval	Ring	54	59	19	19
S2	Spruce	Halden	Drum	50	67	17	20
P	Pine	Kirkenær	Ring	63	56	6	25

S: summer, W: winter.

Supplementary Table S2: The abundance of the 97 compounds in the PCA analysis with the greatest contribution to the PCA separation (PCA loading <3 or <-3) of the different extracts. The compounds are sorted on retention time to demonstrate that pine extracts contain many compounds eluting at low retention times, which are not found in the spruce extracts.

Monoisotopic mass (Da)	Retention Time (min)	S1-S_Ace-Wa	S1-S_Met-Wa	S1-S_Wa	S1-W_Ace-Wa	S1-W_Met-Wa	S1-W_Wa	S2-S_Ace-Wa	S2-S_Met-Wa	S2-S_Wa	S2-W_Ace-Wa	S2-W_Met-Wa	S2-W_Wa	P-S_Ace-Wa	P-S_Met-Wa	P-S_Wa	P-W_Ace-Wa	P-W_Met-Wa	P-W_Wa
560.121	1.24	0.0	0.0	0.0	0.0	0.0	0.0	0.0	0.0	0.0	0.0	0.0	0.0	0.0	0.0	0.0	14.3	14.5	14.5
194.077	1.24	0.0	0.0	0.0	0.0	0.0	0.0	0.0	0.0	0.0	0.0	0.0	0.0	0.0	0.0	0.0	14.6	15.2	15.2
1472.306	1.25	0.0	0.0	0.0	0.0	0.0	0.0	0.0	0.0	0.0	0.0	0.0	0.0	0.0	0.0	0.0	14.7	0.0	15.4
126.032	1.26	0.0	0.0	0.0	0.0	0.0	0.0	0.0	0.0	0.0	0.0	0.0	0.0	0.0	0.0	0.0	15.7	0.0	15.9
144.043	1.26	0.0	0.0	0.0	0.0	0.0	0.0	0.0	0.0	0.0	0.0	0.0	0.0	0.0	0.0	0.0	15.6	15.8	15.8
908.223	1.27	0.0	0.0	0.0	0.0	0.0	0.0	0.0	0.0	0.0	0.0	0.0	0.0	0.0	0.0	0.0	15.0	0.0	15.2
1176.195	1.27	0.0	0.0	0.0	0.0	0.0	0.0	0.0	0.0	0.0	0.0	0.0	0.0	0.0	0.0	0.0	0.0	14.4	15.5
357.128	1.30	0.0	0.0	0.0	0.0	0.0	0.0	0.0	0.0	0.0	0.0	0.0	0.0	0.0	0.0	0.0	15.5	0.0	15.7
429.114	1.43	0.0	0.0	0.0	0.0	0.0	0.0	0.0	0.0	0.0	0.0	0.0	0.0	0.0	0.0	0.0	15.5	15.7	16.4
203.115	1.57	0.0	0.0	0.0	0.0	0.0	0.0	0.0	0.0	0.0	0.0	0.0	0.0	0.0	0.0	0.0	14.6	0.0	14.8
235.142	1.61	0.0	0.0	0.0	0.0	0.0	0.0	0.0	0.0	0.0	0.0	0.0	0.0	0.0	0.0	16.2	15.2	15.2	15.7
736.213	2.84	0.0	0.0	0.0	0.0	0.0	0.0	0.0	0.0	0.0	0.0	0.0	0.0	0.0	0.0	0.0	15.6	0.0	15.3
269.149	3.03	0.0	0.0	0.0	0.0	0.0	0.0	0.0	0.0	0.0	0.0	0.0	0.0	0.0	0.0	0.0	14.7	14.4	14.8
274.103	3.09	0.0	0.0	0.0	0.0	0.0	0.0	0.0	0.0	0.0	0.0	0.0	0.0	0.0	0.0	0.0	16.1	0.0	15.0
363.152	3.09	0.0	0.0	0.0	0.0	0.0	0.0	0.0	0.0	0.0	0.0	0.0	0.0	0.0	0.0	0.0	0.0	15.1	15.4
406.146	3.54	0.0	0.0	0.0	0.0	0.0	0.0	0.0	0.0	0.0	0.0	0.0	0.0	0.0	0.0	0.0	15.0	0.0	15.4
180.079	4.06	0.0	0.0	0.0	0.0	0.0	0.0	0.0	0.0	0.0	0.0	0.0	0.0	0.0	0.0	0.0	15.7	14.4	14.3
385.195	4.46	0.0	0.0	0.0	0.0	0.0	0.0	0.0	0.0	0.0	0.0	0.0	0.0	0.0	0.0	0.0	16.3	16.5	17.0

308.111	4.46	0.0	0.0	0.0	0.0	0.0	0.0	0.0	0.0	0.0	0.0	0.0	0.0	0.0	0.0	0.0	15.1	15.4	15.7
286.106	5.31	0.0	0.0	0.0	0.0	0.0	0.0	0.0	0.0	0.0	0.0	0.0	0.0	0.0	0.0	15.1	15.0	15.6	15.8
486.115	5.31	0.0	0.0	0.0	0.0	0.0	0.0	0.0	0.0	0.0	0.0	0.0	0.0	0.0	0.0	0.0	15.7	15.9	16.4
268.095	5.33	0.0	0.0	0.0	0.0	0.0	0.0	0.0	0.0	0.0	0.0	0.0	0.0	0.0	0.0	0.0	0.0	14.6	14.7
930.736	5.33	0.0	0.0	0.0	0.0	0.0	0.0	0.0	0.0	0.0	0.0	0.0	0.0	0.0	0.0	0.0	0.0	14.3	14.6
1870.684	5.44	0.0	0.0	0.0	0.0	0.0	0.0	0.0	0.0	0.0	0.0	0.0	0.0	0.0	0.0	0.0	0.0	15.2	15.4
463.204	5.46	0.0	0.0	0.0	0.0	0.0	0.0	0.0	0.0	0.0	0.0	0.0	0.0	0.0	0.0	0.0	15.4	0.0	16.0
528.183	5.56	0.0	0.0	0.0	0.0	0.0	0.0	0.0	0.0	0.0	0.0	0.0	0.0	0.0	0.0	0.0	14.7	0.0	14.8
523.230	5.56	0.0	0.0	0.0	0.0	0.0	0.0	0.0	0.0	0.0	0.0	0.0	0.0	0.0	0.0	0.0	14.8	15.4	15.1
435.175	5.70	0.0	0.0	0.0	0.0	0.0	0.0	0.0	0.0	0.0	0.0	0.0	0.0	0.0	0.0	14.8	15.4	16.0	16.2
465.185	5.75	0.0	0.0	0.0	0.0	0.0	0.0	0.0	0.0	0.0	0.0	0.0	0.0	0.0	0.0	0.0	16.9	0.0	17.8
146.037	5.97	0.0	0.0	0.0	0.0	0.0	0.0	0.0	0.0	0.0	0.0	0.0	0.0	15.5	0.0	16.3	17.2	17.3	17.8
338.101	5.98	0.0	0.0	0.0	0.0	0.0	0.0	0.0	0.0	0.0	0.0	0.0	0.0	0.0	0.0	0.0	16.2	0.0	16.7
484.145	6.03	0.0	0.0	0.0	0.0	0.0	0.0	0.0	0.0	0.0	0.0	0.0	0.0	0.0	0.0	0.0	0.0	14.6	14.6
382.104	6.13	0.0	0.0	0.0	0.0	0.0	0.0	0.0	0.0	0.0	0.0	0.0	0.0	0.0	0.0	15.2	16.0	16.1	15.9
361.174	6.14	0.0	0.0	0.0	0.0	0.0	0.0	0.0	0.0	0.0	0.0	0.0	0.0	0.0	0.0	0.0	18.4	18.7	18.5
182.094	6.14	0.0	0.0	0.0	0.0	0.0	0.0	0.0	0.0	0.0	0.0	0.0	0.0	0.0	0.0	0.0	17.0	17.1	16.9
164.083	6.14	0.0	0.0	0.0	0.0	0.0	0.0	0.0	0.0	0.0	0.0	0.0	0.0	0.0	0.0	0.0	0.0	16.3	15.1
366.129	6.14	0.0	0.0	0.0	0.0	0.0	0.0	0.0	0.0	0.0	0.0	0.0	0.0	0.0	0.0	0.0	0.0	17.4	17.2
132.062	6.15	0.0	0.0	0.0	0.0	0.0	0.0	0.0	0.0	0.0	0.0	0.0	0.0	0.0	0.0	0.0	0.0	14.6	14.4
136.053	6.17	0.0	0.0	0.0	0.0	0.0	0.0	0.0	0.0	0.0	0.0	0.0	0.0	0.0	0.0	0.0	16.4	0.0	16.2
493.216	6.27	0.0	0.0	0.0	0.0	0.0	0.0	0.0	0.0	0.0	0.0	0.0	0.0	0.0	0.0	0.0	18.0	18.4	18.1
514.146	6.27	0.0	0.0	0.0	0.0	0.0	0.0	0.0	0.0	0.0	0.0	0.0	0.0	0.0	0.0	0.0	15.9	15.9	15.9
498.171	6.27	0.0	0.0	0.0	0.0	0.0	0.0	0.0	0.0	0.0	0.0	0.0	0.0	0.0	0.0	0.0	18.3	18.3	18.0
196.074	6.83	0.0	0.0	0.0	0.0	0.0	0.0	0.0	0.0	0.0	0.0	0.0	0.0	0.0	0.0	0.0	0.0	17.0	15.5
396.082	6.83	0.0	0.0	0.0	0.0	0.0	0.0	0.0	0.0	0.0	0.0	0.0	0.0	0.0	0.0	0.0	15.4	15.4	14.8
380.109	6.84	0.0	0.0	0.0	0.0	0.0	0.0	0.0	0.0	0.0	0.0	0.0	0.0	0.0	16.2	0.0	17.2	17.4	16.0
493.217	6.90	0.0	0.0	0.0	0.0	0.0	0.0	0.0	0.0	0.0	0.0	0.0	0.0	0.0	0.0	0.0	15.4	16.3	15.4
196.073	7.05	0.0	0.0	0.0	0.0	0.0	0.0	0.0	0.0	0.0	0.0	0.0	0.0	16.9	16.8	14.9	19.3	19.6	18.7

124.054	7.10	0.0	0.0	0.0	0.0	0.0	0.0	0.0	0.0	0.0	0.0	0.0	0.0	0.0	0.0	0.0	14.9	15.3	14.3
218.056	7.10	0.0	0.0	0.0	0.0	0.0	0.0	0.0	0.0	0.0	0.0	0.0	0.0	0.0	0.0	0.0	16.4	16.5	15.7
150.036	7.11	0.0	0.0	0.0	0.0	0.0	0.0	0.0	0.0	0.0	0.0	0.0	0.0	0.0	0.0	0.0	16.2	16.3	15.6
588.576	7.12	0.0	0.0	0.0	0.0	0.0	0.0	0.0	0.0	0.0	0.0	0.0	0.0	0.0	0.0	0.0	0.0	15.7	15.3
507.233	7.12	0.0	0.0	0.0	0.0	0.0	0.0	0.0	0.0	0.0	0.0	0.0	0.0	0.0	0.0	0.0	15.1	15.5	15.5
882.339	7.40	0.0	0.0	0.0	0.0	0.0	0.0	0.0	0.0	0.0	0.0	0.0	0.0	0.0	0.0	0.0	0.0	16.3	15.9
456.140	7.40	0.0	0.0	0.0	0.0	0.0	0.0	0.0	0.0	0.0	0.0	0.0	0.0	0.0	0.0	0.0	15.6	15.3	15.0
361.174	7.50	0.0	0.0	0.0	0.0	0.0	0.0	0.0	0.0	0.0	0.0	0.0	0.0	0.0	0.0	0.0	17.7	18.1	17.3
182.094	7.51	0.0	0.0	0.0	0.0	0.0	0.0	0.0	0.0	0.0	0.0	0.0	0.0	0.0	0.0	0.0	0.0	15.6	14.9
200.104	7.78	0.0	0.0	0.0	0.0	0.0	0.0	0.0	0.0	0.0	0.0	0.0	0.0	0.0	0.0	0.0	15.0	15.2	15.2
180.081	7.80	0.0	0.0	0.0	0.0	0.0	0.0	0.0	0.0	0.0	0.0	0.0	0.0	0.0	0.0	0.0	14.9	14.1	13.9
492.582	7.83	0.0	0.0	0.0	0.0	0.0	0.0	0.0	0.0	0.0	0.0	0.0	0.0	0.0	0.0	0.0	14.6	14.9	14.2
388.171	8.11	0.0	14.7	0.0	0.0	0.0	0.0	0.0	0.0	0.0	0.0	0.0	0.0	0.0	0.0	0.0	15.7	15.7	15.8
182.094	8.17	0.0	0.0	0.0	0.0	0.0	0.0	0.0	0.0	0.0	0.0	0.0	0.0	0.0	0.0	0.0	16.2	16.2	15.6
164.083	8.18	0.0	0.0	0.0	0.0	0.0	0.0	0.0	0.0	0.0	0.0	0.0	0.0	16.0	15.8	0.0	18.4	18.6	17.7
136.052	8.18	0.0	0.0	0.0	0.0	0.0	0.0	0.0	0.0	0.0	0.0	0.0	0.0	0.0	0.0	0.0	18.1	18.3	17.3
417.104	8.18	0.0	0.0	0.0	0.0	0.0	0.0	0.0	0.0	0.0	0.0	0.0	0.0	0.0	0.0	0.0	0.0	15.3	14.9
492.580	8.20	0.0	0.0	0.0	0.0	0.0	0.0	0.0	0.0	0.0	0.0	0.0	0.0	0.0	0.0	0.0	0.0	15.3	14.9
427.206	8.25	0.0	0.0	0.0	0.0	0.0	0.0	0.0	0.0	0.0	0.0	0.0	0.0	0.0	0.0	0.0	14.8	0.0	14.3
671.280	8.41	0.0	0.0	0.0	0.0	0.0	0.0	0.0	0.0	0.0	0.0	0.0	0.0	0.0	0.0	0.0	15.2	15.6	14.4
349.211	8.85	0.0	0.0	0.0	0.0	0.0	0.0	0.0	0.0	0.0	0.0	0.0	0.0	0.0	0.0	14.7	14.8	15.3	14.8
360.156	8.90	0.0	0.0	0.0	0.0	0.0	0.0	0.0	0.0	0.0	0.0	0.0	0.0	0.0	0.0	0.0	16.4	0.0	15.4
530.159	8.98	0.0	0.0	0.0	0.0	0.0	0.0	0.0	0.0	0.0	0.0	0.0	0.0	0.0	0.0	0.0	15.2	15.2	14.8
486.203	9.18	0.0	0.0	0.0	0.0	0.0	0.0	0.0	0.0	0.0	0.0	0.0	0.0	0.0	16.5	0.0	15.0	15.8	15.2
486.208	9.37	0.0	0.0	0.0	0.0	0.0	0.0	0.0	0.0	0.0	0.0	0.0	0.0	0.0	0.0	16.1	16.2	16.5	16.1
778.313	9.42	0.0	0.0	0.0	0.0	0.0	0.0	0.0	0.0	0.0	0.0	0.0	0.0	0.0	0.0	0.0	15.9	16.0	15.4
416.125	9.42	0.0	0.0	0.0	0.0	0.0	0.0	0.0	0.0	0.0	0.0	15.7	0.0	0.0	0.0	15.1	16.5	16.9	16.1
294.097	9.72	0.0	0.0	0.0	0.0	0.0	0.0	0.0	0.0	0.0	0.0	0.0	0.0	0.0	0.0	0.0	15.5	16.0	15.6
494.195	9.80	0.0	0.0	0.0	0.0	0.0	0.0	0.0	0.0	0.0	0.0	0.0	0.0	0.0	0.0	0.0	15.5	0.0	14.8

541.253	9.87	0.0	0.0	0.0	0.0	0.0	0.0	0.0	0.0	0.0	0.0	0.0	0.0	0.0	0.0	0.0	16.1	16.3	15.6
480.161	9.96	0.0	0.0	0.0	0.0	0.0	0.0	0.0	0.0	0.0	0.0	0.0	0.0	17.6	0.0	18.0	19.3	19.1	19.0
475.205	9.97	0.0	0.0	0.0	0.0	0.0	0.0	0.0	0.0	0.0	0.0	0.0	0.0	18.0	18.3	18.5	19.8	20.3	20.1
496.137	10.03	0.0	0.0	0.0	0.0	0.0	0.0	0.0	0.0	0.0	0.0	0.0	0.0	0.0	0.0	0.0	17.2	17.5	16.9
445.195	10.03	0.0	0.0	0.0	0.0	0.0	0.0	0.0	0.0	0.0	0.0	0.0	0.0	0.0	0.0	0.0	17.0	17.6	17.5
459.211	10.07	0.0	0.0	0.0	0.0	0.0	0.0	0.0	0.0	0.0	0.0	0.0	0.0	0.0	0.0	0.0	15.9	16.8	15.9
494.179	10.22	0.0	0.0	0.0	0.0	0.0	0.0	0.0	0.0	0.0	15.4	0.0	0.0	0.0	0.0	0.0	15.6	15.4	15.5
480.161	10.24	0.0	0.0	0.0	0.0	0.0	0.0	0.0	0.0	0.0	0.0	0.0	0.0	0.0	0.0	0.0	0.0	17.3	17.2
489.221	10.25	0.0	0.0	0.0	0.0	0.0	0.0	0.0	0.0	0.0	0.0	0.0	0.0	0.0	0.0	0.0	17.6	18.2	17.8
475.205	10.25	0.0	0.0	0.0	0.0	0.0	0.0	0.0	0.0	0.0	0.0	0.0	0.0	0.0	0.0	0.0	0.0	18.7	18.5
218.094	10.30	0.0	16.0	0.0	0.0	0.0	0.0	0.0	0.0	0.0	0.0	0.0	0.0	0.0	0.0	0.0	16.1	16.6	15.4
746.327	10.86	0.0	0.0	0.0	0.0	0.0	0.0	0.0	0.0	0.0	0.0	0.0	0.0	0.0	0.0	0.0	16.1	16.4	15.8
400.129	10.88	0.0	0.0	0.0	0.0	0.0	0.0	0.0	0.0	0.0	0.0	0.0	0.0	0.0	0.0	0.0	15.2	0.0	15.3
180.083	10.96	0.0	0.0	0.0	0.0	0.0	0.0	0.0	0.0	0.0	0.0	0.0	0.0	0.0	0.0	0.0	0.0	14.2	14.2
300.137	11.33	0.0	0.0	0.0	0.0	0.0	0.0	0.0	0.0	0.0	0.0	0.0	0.0	0.0	0.0	0.0	16.3	0.0	15.7
438.227	11.60	0.0	0.0	0.0	14.8	0.0	0.0	0.0	0.0	0.0	0.0	0.0	0.0	0.0	0.0	0.0	15.6	15.7	14.7
524.241	11.67	0.0	0.0	0.0	0.0	0.0	0.0	0.0	0.0	0.0	0.0	0.0	0.0	15.0	0.0	0.0	15.9	16.4	15.2
358.141	12.41	0.0	0.0	0.0	0.0	0.0	0.0	0.0	0.0	0.0	0.0	0.0	0.0	0.0	17.5	0.0	17.7	17.8	15.8
1293.070	14.89	0.0	0.0	0.0	0.0	0.0	0.0	0.0	0.0	0.0	0.0	0.0	0.0	0.0	0.0	0.0	0.0	15.9	14.9
414.205	16.38	0.0	0.0	0.0	0.0	0.0	0.0	0.0	0.0	0.0	0.0	0.0	0.0	0.0	0.0	0.0	15.8	0.0	15.2
4700.622	18.51	0.0	0.0	0.0	0.0	0.0	0.0	0.0	0.0	0.0	0.0	0.0	0.0	0.0	0.0	0.0	0.0	15.6	15.7

S1: spruce, sawmill, ring debarking; S2: spruce, pulp mill, drum debarking; P: pine, sawmill, ring debarking, collected during summer (S) and winter (W) seasons.
Each of the bark samples was extracted using water (Wa), acetone-water (Ace-Wa), or methanol-water (Met-Wa) as the solvent.

Supplementary Table S3: MS-abundance (total ion counts, tic) of CT mono, di- and trimers, identified by a targeted search for the respective masses. All are procyanidins (catechin or epicatechin as monomeric units). Any prodelphinidins were below the detection levels.

Extract	Monomer	Dimer	Trimer
S1-S_Ace-Wa		37,407	
S1-S_Met-Wa		59,919	
S1-S_Wa		61,031	
S1-W_Ace-Wa	23,614	74,302	
S1-W_Met-Wa		50,777	
S1-W_Wa			
S2-S_Ace-Wa			
S2-S_Met-Wa			
S2-S_Wa			
S2-W_Ace-Wa			
S2-W_Met-Wa			
S2-W_Wa		222,519	
P-S_Ace-Wa			126,037
P-S_Met-Wa	142,059	470,652	237,199
P-S_Wa	161,001		82,056
P-W_Ace-Wa		142,131	
P-W_Met-Wa	69,103	74,168	66,362
P-W_Wa		37,407	

S1: spruce, sawmill, ring debarking; S2: spruce, pulp mill, drum debarking; P: pine, sawmill, ring debarking, collected during summer (S) and winter (W) seasons. Each of the bark samples was extracted using water (Wa), acetone-water (Ace-Wa), or methanol-water (Met-Wa) as the solvent.

Supplementary Table S4: Pearson correlation between abundance (log2) of each of the LC-MS determined masses in the extracts and the estimated egg hatching IC50 values of *Trichostrongylus colubriformis* and *Trichostrongylus colubriformis*

Pearson correlation between abundance (log2) of each of the LC-MS determined masses in the extracts and the estimated egg hatching IC50 values of *Trichostrongylus colubriformis*

Trichostrongylus colubriformis

		Mass	Retention time	Abundance (log2)				Pearson correlation	
	n	Da	min	Mean	Std	Minimum	Maximum	r	P-value
All extracts	18	CT		89,31	33,66	16,70	153,30	-0,53835	0,0212
	18	662,5327	18,37	4,62	2,12895	0	5,66	-0,54158	0,0203
Methanol and	12	CT		103,425	55,16113	67,7	153,3	-0,32025	0,3102
acetone extract	12	164,0832	8,2	1,72417	2,55807	0	5,6	-0,82149	0,001
	12	196,0733	7,1	1,82	2,69864	0	5,89	-0,82086	0,0011
	12	379,1993	10,9	1,81167	2,68418	0	5,87	-0,8193	0,0011
	12	475,2052	10,0	1,91583	2,8353	0	6,11	-0,81787	0,0012
	12	146,0367	6,0	1,25417	2,2725	0	5,21	-0,72473	0,0077
	12	650,5853	18,4	6,4825	0,29918	5,76	6,78	-0,72422	0,0077
	12	480,1614	10,0	1,40417	2,54295	0	5,8	-0,72394	0,0078
	12	524,2406	11,7	1,1875	2,15015	0	4,95	-0,72261	0,0079
	12	362,1727	10,9	1,4225	2,57781	0	5,96	-0,71785	0,0086
	12	380,1093	6,8	1,27667	2,31113	0	5,25	-0,71584	0,0088
	12	518,1792	8,4	1,15917	2,09709	0	4,67	-0,71262	0,0093
	12	358,1414	12,4	1,33083	2,40765	0	5,36	-0,71255	0,0093
	12	486,2034	9,2	1,1875	2,15033	0	4,96	-0,70542	0,0104
	12	332,1989	13,0	1,78583	2,63925	0	5,58	-0,70025	0,0112
	12	494,1794	10,2	1,165	2,10759	0	4,69	-0,6939	0,0123
	12	298,1930	13,6	2,1375	2,64974	0	5,63	-0,6723	0,0166
	12	359,1423	1,3	2,59333	2,72113	0	5,74	-0,65869	0,0198
	12	638,6221	17,8	6,91417	0,2539	6,49	7,2	-0,64955	0,0223
	12	375,1680	12,4	1,67583	2,48991	0	5,36	-0,64894	0,0224
	12	456,1397	7,4	0,77417	1,80817	0	4,69	-0,63738	0,0258
	12	269,1494	3,0	0,73083	1,70692	0	4,42	-0,63735	0,0258
	12	498,1713	6,3	0,91667	2,14087	0	5,5	-0,63721	0,0258
	12	396,0823	6,8	0,775	1,81001	0	4,65	-0,63721	0,0258
	12	514,1456	6,3	0,79917	1,86645	0	4,8	-0,63719	0,0258
	12	182,0939	8,2	0,81417	1,90148	0	4,89	-0,63719	0,0258
	12	778,3130	9,4	0,79917	1,86645	0	4,8	-0,63719	0,0258
	12	530,1587	9,0	0,7625	1,78082	0	4,58	-0,63719	0,0258
	12	235,1416	1,6	0,76333	1,78277	0	4,59	-0,63716	0,0259
	12	182,0938	6,1	0,8575	2,0027	0	5,16	-0,63715	0,0259
	12	150,0356	7,1	0,81417	1,90149	0	4,9	-0,63714	0,0259
	12	218,0558	7,1	0,825	1,9268	0	4,97	-0,63712	0,0259
	12	382,1037	6,1	0,80583	1,88205	0	4,86	-0,63709	0,0259
	12	136,0524	8,2	0,91417	2,13509	0	5,52	-0,63706	0,0259
	12	486,1148	5,3	0,79	1,84509	0	4,77	-0,63706	0,0259
	12	429,1143	1,4	0,785	1,83341	0	4,74	-0,63706	0,0259
	12	496,1373	10,0	0,87083	2,03388	0	5,26	-0,63705	0,0259
	12	144,0431	1,3	0,78583	1,83537	0	4,75	-0,63703	0,0259
	12	385,1952	4,5	0,82167	1,91908	0	4,97	-0,63701	0,0259
	12	541,2534	9,9	0,81333	1,89961	0	4,92	-0,63701	0,0259
	12	560,1212	1,2	0,7225	1,68746	0	4,37	-0,63701	0,0259
	12	361,1736	6,1	0,93	2,17212	0	5,63	-0,63699	0,0259
	12	200,1042	7,8	0,75833	1,77117	0	4,59	-0,63699	0,0259
	12	486,2078	9,4	0,81917	1,91326	0	4,96	-0,63698	0,0259
	12	746,3274	10,9	0,81417	1,90158	0	4,93	-0,63698	0,0259
	12	308,1112	4,5	0,76333	1,78289	0	4,63	-0,63693	0,0259
	12	361,1738	7,5	0,89833	2,09827	0	5,46	-0,63687	0,0259
	12	671,2796	8,4	0,77167	1,80241	0	4,69	-0,63687	0,0259
	12	507,2329	7,1	0,76583	1,78882	0	4,66	-0,63683	0,026
	12	489,2205	10,3	0,9	2,1023	0	5,49	-0,63675	0,026
	12	294,0972	9,7	0,78917	1,84345	0	4,82	-0,63671	0,026
	12	286,1063	5,3	0,7675	1,79286	0	4,69	-0,63669	0,026
	12	445,1947	10,0	0,86667	2,02455	0	5,3	-0,63666	0,026
	12	435,1746	5,7	0,78917	1,84354	0	4,83	-0,63663	0,026
	12	194,0774	1,2	0,74667	1,74426	0	4,57	-0,63663	0,026
	12	523,2295	5,6	0,7575	1,76971	0	4,65	-0,63652	0,026

	12	459,2107	10,1	0,82083	1,91779	0	5,05	-0,63643	0,0261
	12	493,2168	6,9	0,79417	1,8558	0	4,91	-0,63621	0,0261
	12	400,1495	9,4	4,395	2,06811	0	5,73	-0,6209	0,0312
	12	697,5861	18,4	4,755	2,22219	0	5,82	-0,61965	0,0316
	12	294,2185	13,4	1,19417	2,16103	0	4,92	-0,61546	0,0331
	12	136,0526	3,0	1,59833	2,36292	0	4,97	-0,60781	0,036
	12	367,2357	15,4	1,28333	2,32542	0	5,43	-0,59836	0,0398
	12	690,4109	15,4	1,29333	2,34229	0	5,41	-0,59705	0,0404
	12	866,2068	8,3	1,17083	2,1196	0	4,88	-0,5943	0,0416
	12	866,2049	6,9	1,18833	2,15265	0	5,04	-0,59284	0,0422
	12	320,1996	15,0	1,22667	2,22095	0	5,13	-0,59083	0,0431
	12	369,2508	14,0	2,03	2,51029	0	5,04	-0,58978	0,0435
	12	316,2038	15,9	2,13667	2,64489	0	5,44	-0,57922	0,0484
	12	674,6676	18,3	2,1925	2,71065	0	5,4	-0,57892	0,0486
Methanol extract		CT		95,36667	27,63271	62	215	-0,32157	0,5343
	6	200,1600	17,89	17,1782	0,84439	16,40431	18,50354	-0,99325	<.0001
	6	498,1700	7,14	6,01662	9,38394	0	19,76674	-0,93298	0,0066
	6	136,0500	9,36	6,14625	9,56884	0	19,93825	-0,93202	0,0068
	6	150,0300	8,19	5,60532	8,71302	0	17,94455	-0,93076	0,007
	6	196,0700	8,09	6,64857	10,3324	0	21,23991	-0,93054	0,0071
	6	466,1200	10,45	5,69364	8,84533	0	18,1272	-0,93016	0,0071
	6	146,0400	7,13	5,88432	9,14039	0	18,70904	-0,93	0,0072
	6	360,1600	11,73	5,89973	9,16304	0	18,72985	-0,92983	0,0072
	6	132,0600	9,39	8,383	9,24905	0	18,77808	-0,92939	0,0073
	6	475,2100	10,59	5,8411	9,068	0	18,45107	-0,92922	0,0073
	6	470,1400	6,29	5,97938	9,27898	0	18,793	-0,92858	0,0075
	6	226,1000	17,37	6,20301	9,62438	0	19,45014	-0,92826	0,0075
	6	480,1600	10,58	6,67246	10,35177	0	20,89326	-0,92807	0,0076
	6	578,2100	10,74	5,80522	9,00469	0	18,12826	-0,9277	0,0077
	6	154,0600	6,18	5,29601	8,21484	0	16,53804	-0,92769	0,0077
	6	380,1100	7,6	6,17629	9,57731	0	19,18669	-0,92694	0,0078
	6	230,0800	1,51	5,39339	8,36128	0	16,67579	-0,92623	0,008
	6	274,1600	13,95	5,3435	8,28314	0	16,48649	-0,9259	0,008
	6	524,2400	12,19	5,34592	8,28682	0	16,49079	-0,92587	0,008
	6	342,0100	10,28	5,54053	8,58624	0	16,974	-0,92501	0,0082
	6	256,1100	17,2	5,35063	8,28992	0	16,22959	-0,92303	0,0087
	6	262,1200	10,43	5,33155	8,25968	0	16,05098	-0,92166	0,009
	6	332,1300	9,14	5,25442	8,14015	0	15,80135	-0,92052	0,0092
	6	164,0800	10,45	5,42966	8,41175	0	16,37053	-0,92001	0,0093
	6	590,1800	12,69	5,36512	8,31259	0	16,29711	-0,91847	0,0097
	6	218,1700	10,1	5,5139	8,5433	0	16,7679	-0,91823	0,0098
	6	550,1800	12,69	5,47733	8,48677	0	16,66936	-0,91807	0,0098
	6	646,0600	10,27	5,23914	8,11802	0	15,97044	-0,91771	0,0099
	6	510,2100	11,82	5,45265	8,44893	0	16,62695	-0,91764	0,0099
	6	632,0700	10,27	5,11713	7,93017	0	15,68124	-0,91654	0,0102
	6	276,2400	17,7	5,55394	8,61358	0	17,29966	-0,91262	0,0111
	6	200,1600	16,81	5,62436	8,72621	0	17,62553	-0,91105	0,0115
	6	332,0900	11,2	5,15171	7,99577	0	16,22329	-0,90974	0,0119
	6	332,2000	13,96	5,97243	9,25604	0	18,32534	-0,90974	0,0119
	6	202,1700	15,87	5,57106	8,64969	0	17,62019	-0,90854	0,0122
	6	448,1200	10,33	5,54899	8,62091	0	17,67283	-0,90656	0,0127
	6	152,1200	5,08	8,16086	8,94733	0	16,97628	-0,90193	0,014
	6	208,1300	6,54	8,13605	8,91307	0	16,4337	-0,89791	0,0151
	6	538,1800	8,91	7,88264	8,638	0	15,97482	-0,89395	0,0163
	6	298,1900	17,96	10,30319	11,2912	0	21,12932	-0,89172	0,017
	6	154,0300	4,91	8,15887	8,93948	0	16,63757	-0,88891	0,0178
	6	117,0800	1,28	19,33662	0,3936	18,82809	19,96309	-0,88079	0,0205
	6	304,0600	10,27	19,14102	1,30414	17,63549	20,9364	-0,8641	0,0264
	6	578,2100	11,23	18,86001	0,29846	18,57104	19,36506	-0,84823	0,0328
	6	114,1000	15,46	19,25991	0,05619	19,21382	19,36658	-0,82287	0,0443
	6	300,1400	12,06	15,89289	0,83543	14,94073	17,19186	-0,81138	0,05

CT = condensed tannins

Pearson correlation between abundance (log2) of each of the LC-MS determined masses in the extracts and the estimated egg hatching IC50 values of *Teladorsagia circumcincta*

Teladorsagia circumcincta

		Mass	Retention time	Abundance (log2)				Pearson correlation	
	n	Da	min	Mean	Std	Minimum	Maximum	r	P-value
All extracts	18	CT		89,3	33,7	16,7	153,3	-0,140	0,579
	18	148,0172	17,93	4,722	1,180	0	5,09	-0,998	<.0001
	18	618,5189	18,37	4,941	1,799	0	5,66	-0,684	0,002
	18	278,1543	17,93	4,094	1,885	0	5,04	-0,541	0,021
	18	256,1308	7,84	4,708	2,185	0	6,08	-0,536	0,022
Methanol and acetone	12	CT		103,425	55,161	67,7	153,3	-0,185	0,565
	12	386,1342	9,17	4,381	1,421	0	5,39	-0,977	<.0001
	12	390,2778	18,07	5,047	1,590	0	5,62	-0,970	<.0001
	12	400,1495	9,44	4,395	2,068	0	5,73	-0,824	0,001
	12	697,5861	18,39	4,755	2,222	0	5,82	-0,629	0,028
	12	638,6221	17,83	6,914	0,254	6,49	7,2	-0,602	0,038

CT = condensed tannins

Supplementary Table S5: Mass spectrum (black bar) from positive ionization (ESI+) of selected compounds. The red bars are the theoretical isotopic distribution of the suggested formulas. Thus, molecular formula that fits the observed mass spectrum could be predicted for these compounds.

Neutral mass (Da)	Suggested molecular formula	Retention time (min)	Mass spectrum (M+H) with predicted isotope distribution
164.0832	$C_{10}H_{12}O_2$	8.2	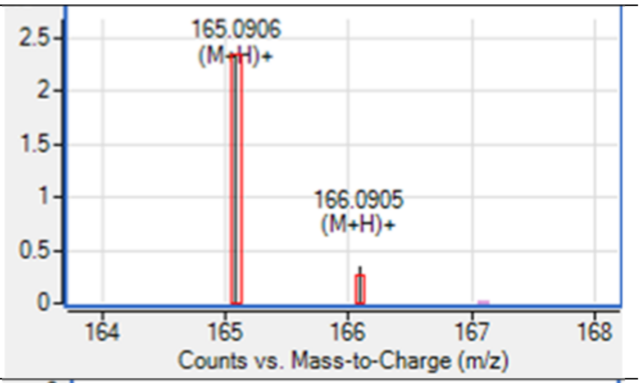
200.1557	$C_{15}H_{20}$	15.4	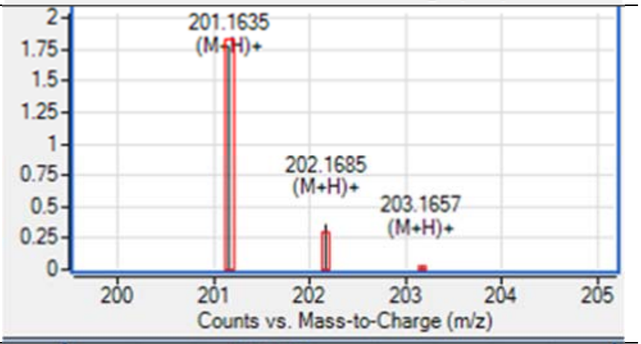
226.1004	$C_{15}H_{14}O_2$	16.2	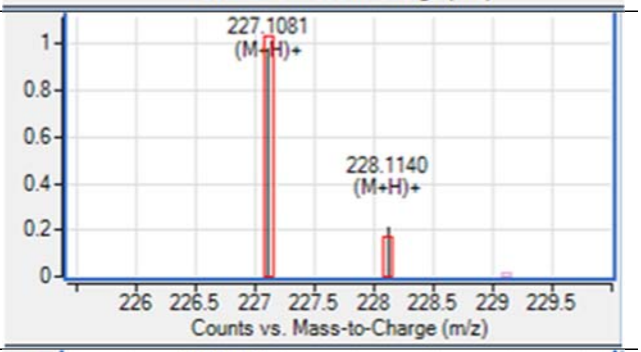
300.2089	$C_{20}H_{28}O_2$	17.6	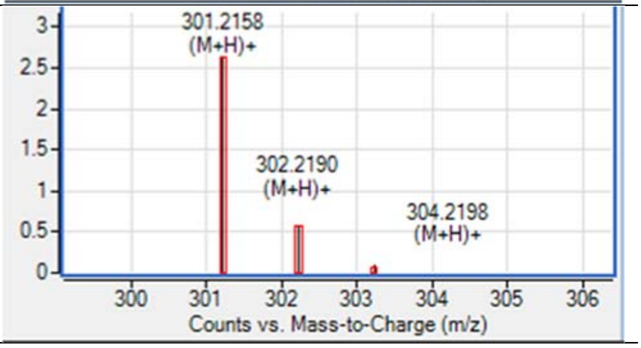

Figures

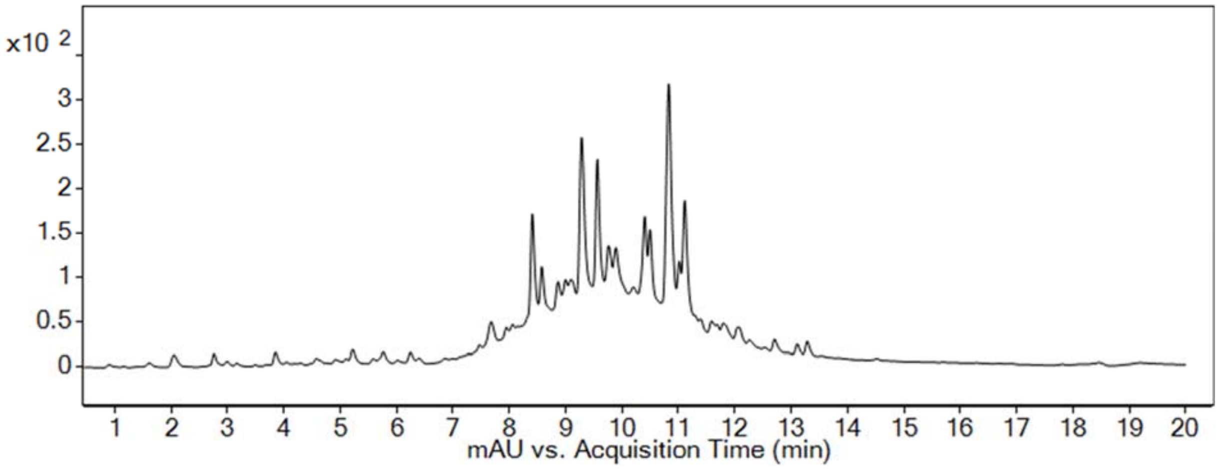

a) S1-W extracted with methanol-water

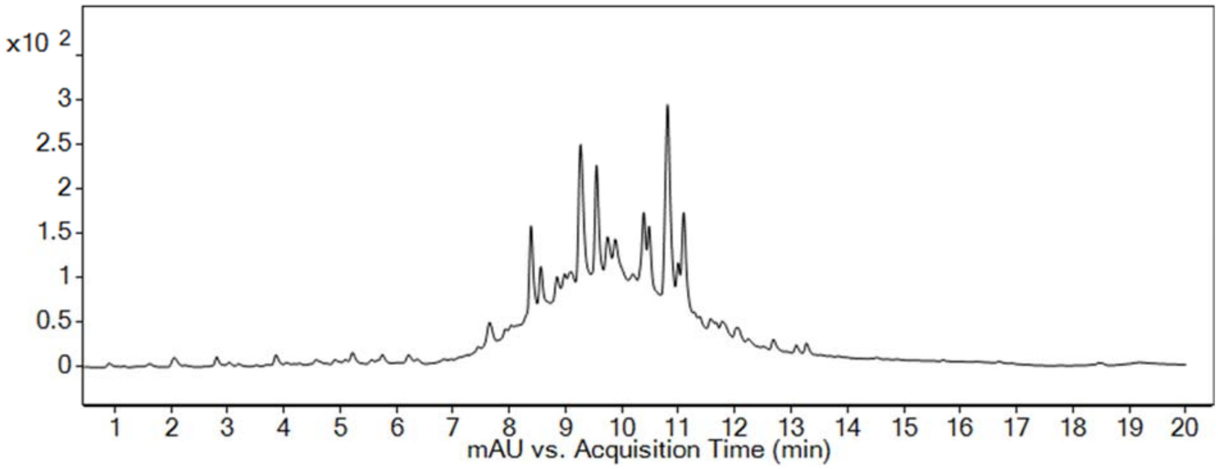

b) S1-W extracted with acetone-water

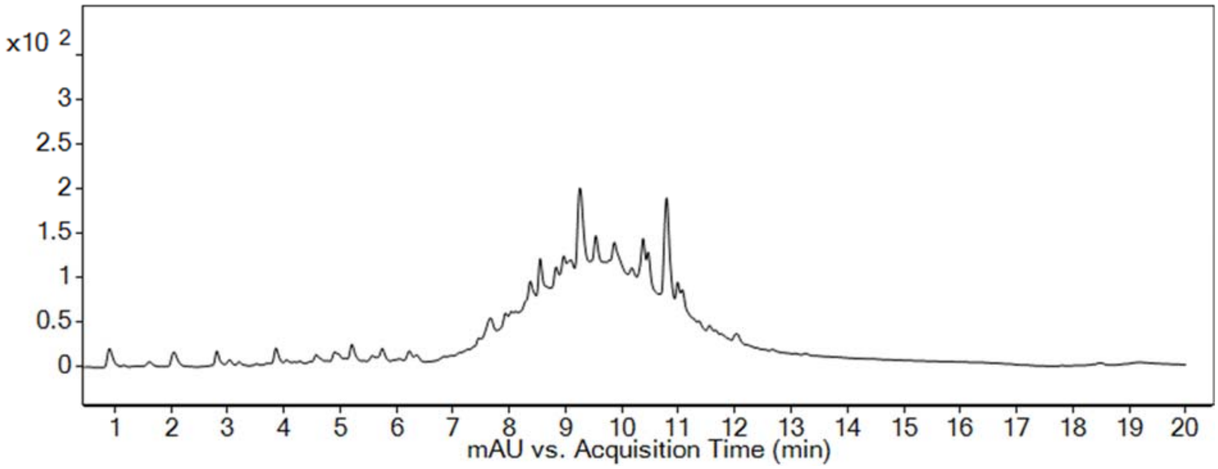

c) S1-W extracted with water

(Figure legend, see next page)

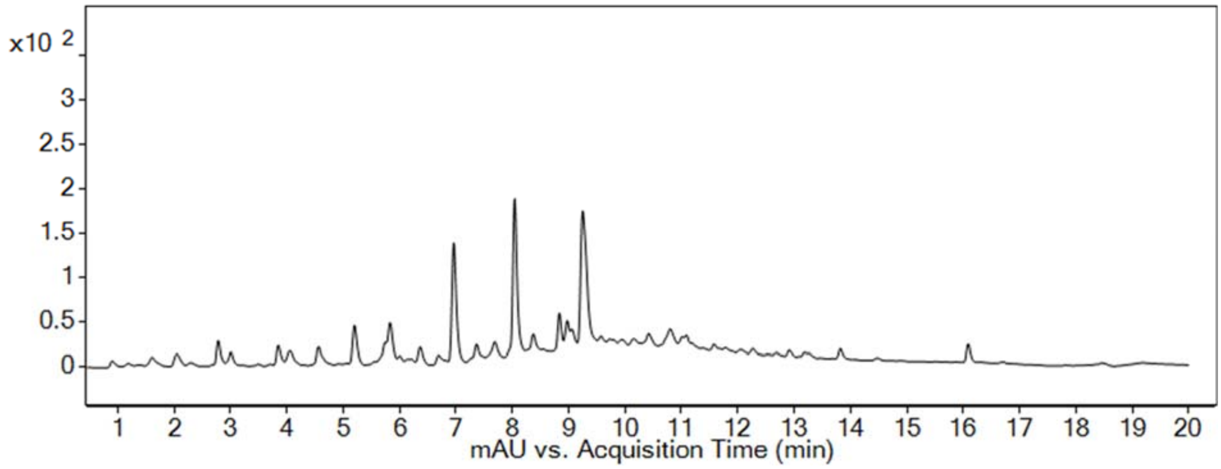

d) P-W extracted with methanol-water

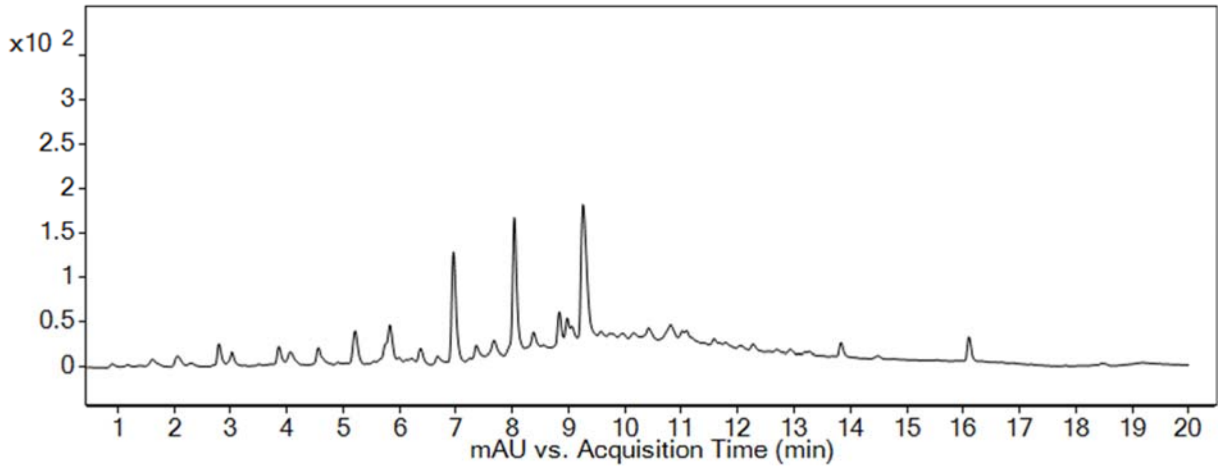

e) P-W extracted with acetone-water

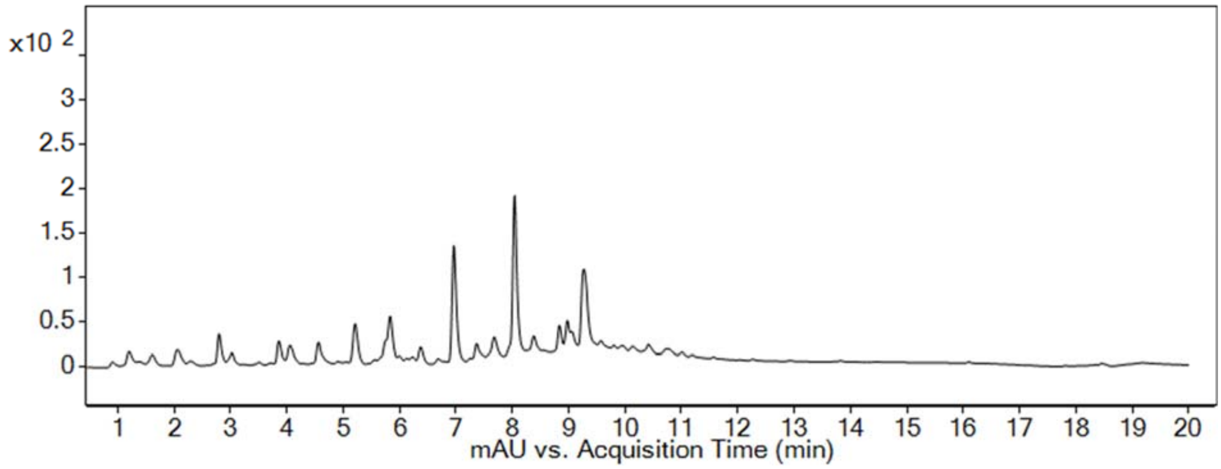

f) P-W extracted with water

Supplementary Fig. S1: UV280 nm chromatogram of spruce (S1-W) and pine (P-W) extracted with methanol-water, acetone-water and water.

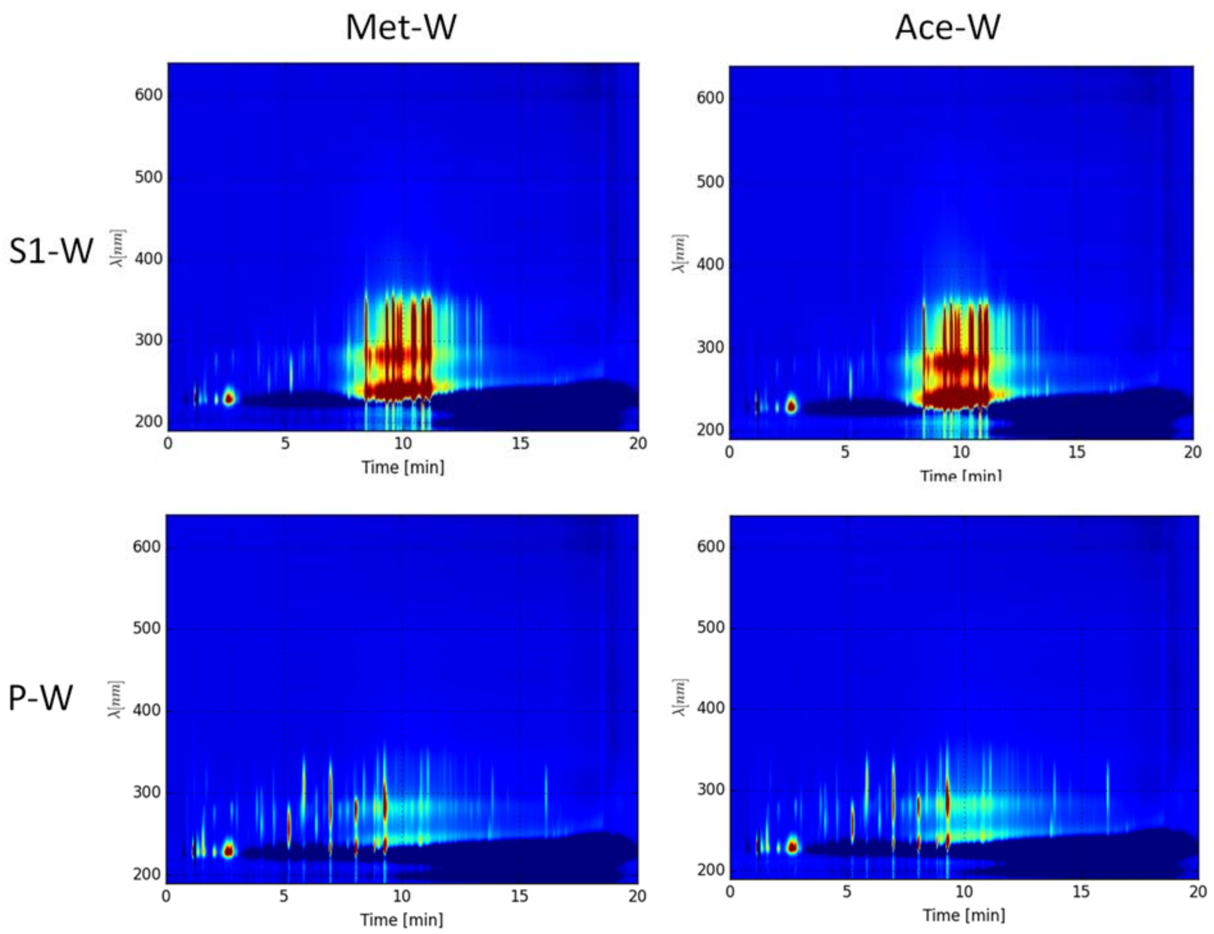

Supplementary Fig. S2: LC-DAD-isoplots of S1-W (spruce) and P-W (pine) extracted with methanol-water (Met-W) and acetone-water (Ace-W).

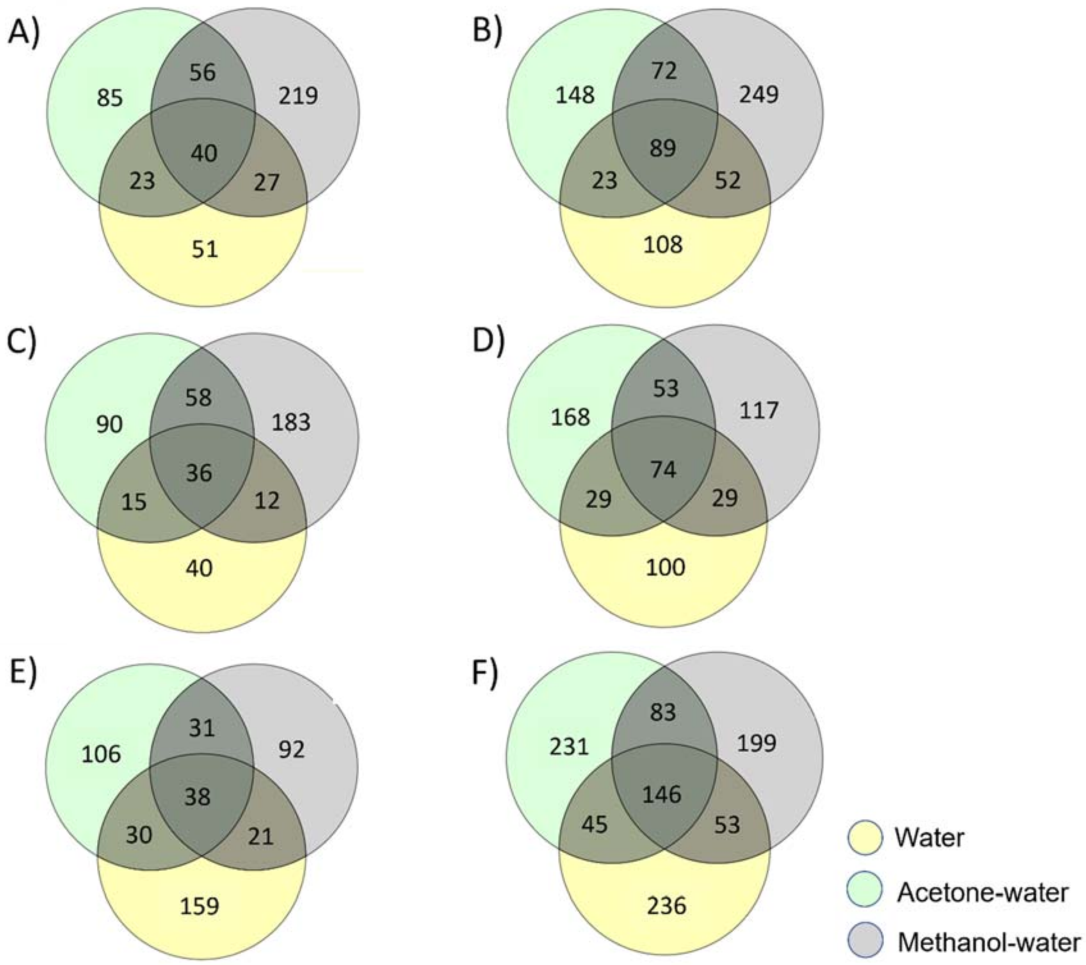

Supplementary Fig. S3: Venn diagram displaying the number of compounds (entities) detected with high resolution mass spectrometry in the bark batches extracted using water, acetone-water and methanol-water. A: S1-S, B: S1-W, C: S2-S, D: S2-W, E: P-S and F: P-W.

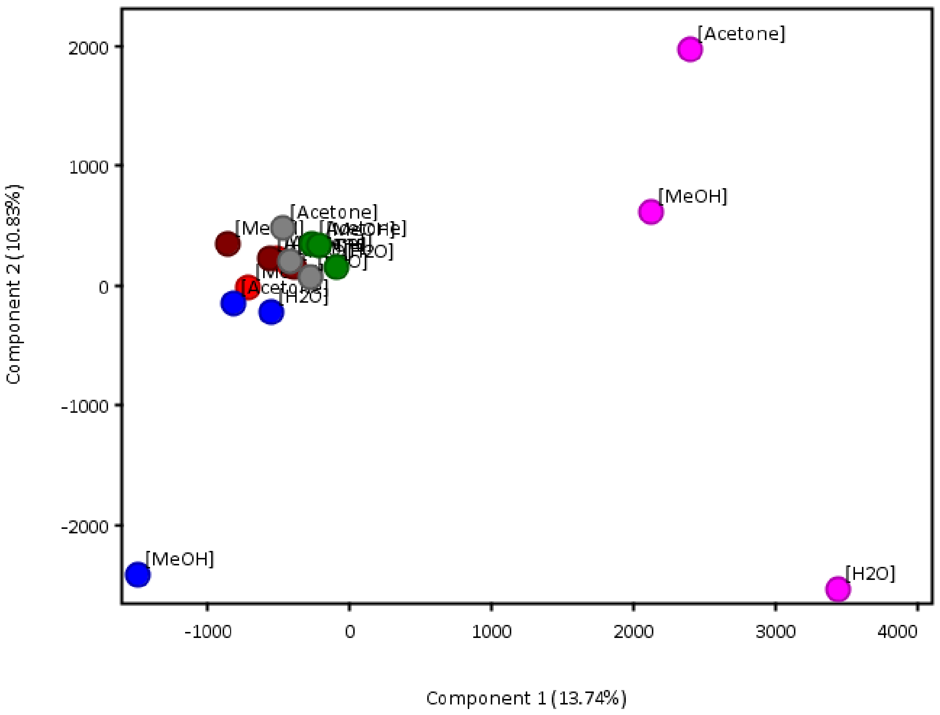

Supplementary Fig. S4: Principal component analysis of all 2299 compounds that were detected in the 18 bark extracts from mass-spectrometry. Extract colour codes: spruce 1 (sawmill, ring debarking) from summer (red) and winter (blue); spruce 2 (pulp mill, drum debarking) from summer (brown) and winter (grey); and pine (sawmill, ring debarking) from summer (green) and winter (pink). Extraction solvents are noted next to the dots; water (H2O), methanol (MeOH) and Acetone.

A)

Monoisotopic mass (Da)	Rt [min]	Molecular formula	Putative annotation	Compound class	Occurrence in (species)	Correlation analysis			
						Me		Me_Ac	
						r	p-value	r	p-value
164.0832	8.18	C10H12O2	Eugenol, thujaplicin, thymoquinone	Fenol, monoterpenoid	Both	-0.92	0.01	-0.82	0.001
196.0733	7.05	C10H12O4	Acetosyringone, atraric acid, brevifolin	Benzoic acid derivative	Pine	-0.93	0.01	-0.82	0.001
200.1557	15.4	C15H20	Corocalene, calacorene	Sesquiterpenoids	Both	-0.99	<.0001		
226.1004	16.24	C15H14O2	Pinosylvin monomethyl ether	Stilbens	Pine	-0.93	0.01		
300.2089	16.7	C20H28O2	Several options		Both			-0.71	0.005
379.1993	10.86	C24H29NOS	No good hits on natural compounds		Pine			-0.82	0.001
380.1093	6.8	C22H20O4S	Several options	Substituted furan	Pine	-0.93	0.01	-0,72	0.009

B)

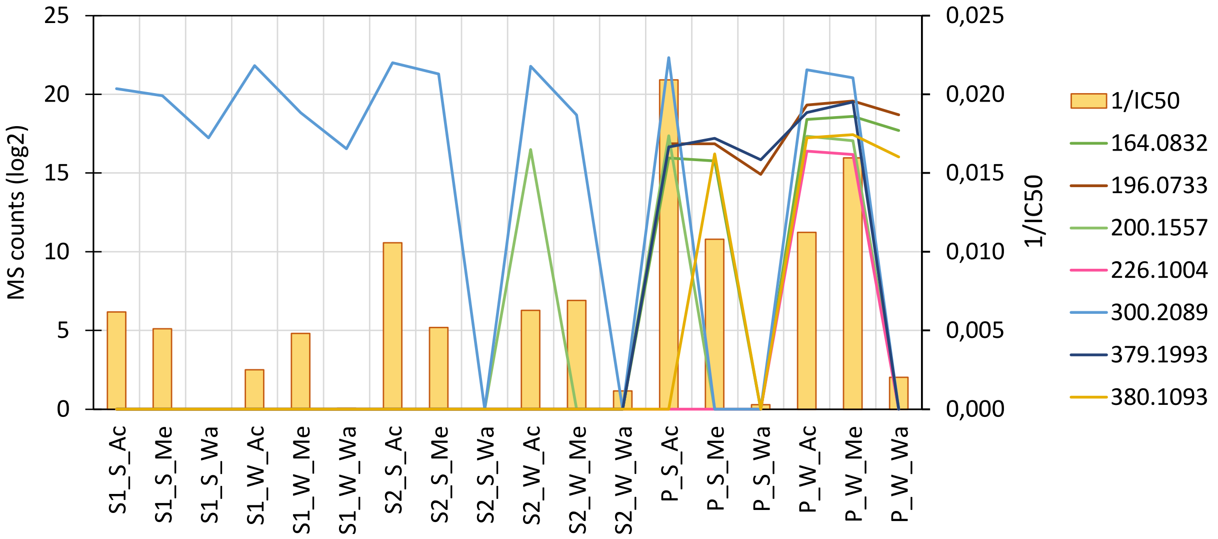

Supplementary Fig. S5: Selected masses where the abundance in the extracts from two independent MS-analyses correlated with the inhibition of egg hatching in *T. colubriformis* (low p-value/high r-value). **A):** Molecular formulas for the selected masses generated based on the molecular mass and MS isotopic distribution, and possible annotations based on data base search. **B)** The abundance of the selected compounds (MS_counts) plotted against *T. colubriformis* 1/IC50 to visualize how abundance correlated with bioactivity in EHA. All extracts were analysed using an Electrospray Ionization (ESI) Source, while the Met-Wa ("Me") extracts were additionally analysed using a more sensitive "Jet Stream" ion source. The masses observed in the two analyses ("Me": Met-Wa and "Me_Ac": Met-Wa and Ace-Wa) were correlated with bioactivity against *T. colubriformis* using Pearson correlation. Masses that correlated with the inhibition of *T. colubriformis* were manually evaluated by comparing the mass profile (abundance vs sample) with the bioactivity (1/IC50).
